# Cigarette Smoking and E-cigarette Use Induce Shared DNA Methylation Changes Linked to Carcinogenesis

**DOI:** 10.1158/0008-5472.CAN-23-2957

**Published:** 2024-03-19

**Authors:** Chiara Herzog, Allison Jones, Iona Evans, Janhavi R. Raut, Michal Zikan, David Cibula, Andrew Wong, Hermann Brenner, Rebecca C. Richmond, Martin Widschwendter

**Affiliations:** 1 European Translational Oncology Prevention and Screening (EUTOPS) Institute, Universität Innsbruck, Innsbruck, Austria.; 2 Research Institute for Biomedical Aging, Universität Innsbruck, Innsbruck, Austria.; 3 Department of Women's Cancer, UCL EGA Institute for Women's Health, University College London, London, United Kingdom.; 4 Division of Clinical Epidemiology and Aging Research, German Cancer Research Center (DKFZ), Heidelberg, Germany.; 5 German Cancer Consortium (DKTK), German Cancer Research Center (DKFZ), Heidelberg, Germany.; 6 Department of Gynecology and Obstetrics, First Faculty of Medicine and Hospital Na Bulovce, Charles University in Prague, Prague, Czech Republic.; 7 Gynecologic Oncology Center, Department of Obstetrics and Gynecology, First Faculty of Medicine, Charles University in Prague, General University Hospital in Prague, Prague, Czech Republic.; 8 MRC Unit for Lifelong Health and Ageing, Institute of Cardiovascular Science, University College London, London, United Kingdom.; 9 MRC Integrative Epidemiology Unit at the University of Bristol, Bristol, United Kingdom.; 10 Population Health Sciences, Bristol Medical School, University of Bristol, Bristol, United Kingdom.; 11 Department of Women's and Children's Health, Karolinska Institutet, Stockholm, Sweden.

## Abstract

Tobacco use is a major modifiable risk factor for adverse health outcomes, including cancer, and elicits profound epigenetic changes thought to be associated with long-term cancer risk. While electronic cigarettes (e-cigarettes) have been advocated as harm reduction alternatives to tobacco products, recent studies have revealed potential detrimental effects, highlighting the urgent need for further research into the molecular and health impacts of e-cigarettes. Here, we applied computational deconvolution methods to dissect the cell- and tissue-specific epigenetic effects of tobacco or e-cigarette use on DNA methylation (DNAme) in over 3,500 buccal/saliva, cervical, or blood samples, spanning epithelial and immune cells at directly and indirectly exposed sites. The 535 identified smoking-related DNAme loci [cytosine-phosphate-guanine sites (CpG)] clustered into four functional groups, including detoxification or growth signaling, based on cell type and anatomic site. Loci hypermethylated in buccal epithelial cells of smokers associated with *NOTCH1*/*RUNX3*/growth factor receptor signaling also exhibited elevated methylation in cancer tissue and progressing lung carcinoma *in situ* lesions, and hypermethylation of these sites predicted lung cancer development in buccal samples collected from smokers up to 22 years prior to diagnosis, suggesting a potential role in driving carcinogenesis. Alarmingly, these CpGs were also hypermethylated in e-cigarette users with a limited smoking history. This study sheds light on the cell type–specific changes to the epigenetic landscape induced by smoking-related products.

**Significance:**

The use of both cigarettes and e-cigarettes elicits cell- and exposure-specific epigenetic effects that are predictive of carcinogenesis, suggesting caution when broadly recommending e-cigarettes as aids for smoking cessation.

Watch the interview with Chiara Herzog, PhD, recipient of the 2026 *Cancer Research* Early Career Award: https://vimeo.com/1208910914

## Graphical Abstract

**Figure:**
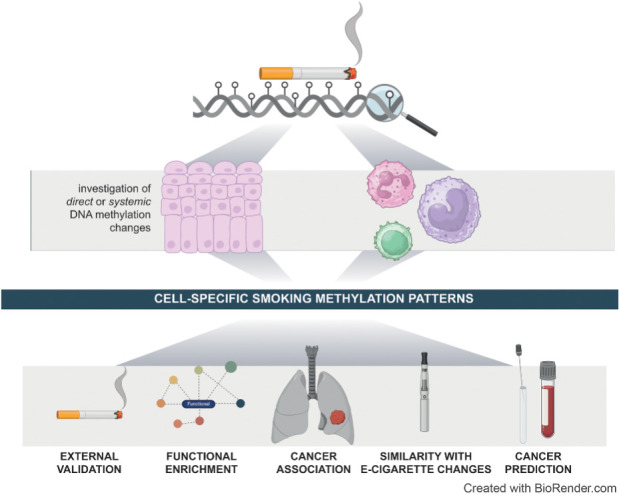

## Introduction

Tobacco usage elicits a spectrum of detrimental effects spanning cellular, organ, and systemic levels, encompassing DNA damage, inflammation, oxidative stress, and epigenetic alterations (1), and is a known modifiable contributor to adverse health outcomes. Exposure to the 7,000 chemicals in cigarettes (2) has been estimated to have caused 7.69 million deaths globally in 2019, with numbers projected to increase in the ensuing decades (3).

Seeking harm reduction, alternatives to smoking such as smokeless noncombustible tobacco use (4) and electronic cigarettes (e-cigarettes; ref. 5) that vaporize a liquid solution often containing nicotine and various other chemicals have emerged. Despite widespread endorsement by Public Health England, who have advocated electronic cigarettes (e-cigarettes) as “95% less harmful” than combustible cigarettes (6), recent studies have highlighted potential drawbacks, including the induction of endothelial dysfunction (7) and DNA damage (8), underscoring the urgency for further research into molecular changes and long-term health impacts of e-cigarettes (9). However, the relative novelty of e-cigarettes and the fact that many e-cigarette users (“vapers”) are also former smokers renders this task complex and studies with several decades of follow-up would be required to investigate the impact of e-cigarette use on cancer risk if incidence were the primary outcome. Biomarkers could represent an attractive strategy to evaluate their impact in the absence of such long-term studies. The majority of existing biomarker studies for e-cigarette use have thus far focused only on acute impacts. Some of these studies have found e-cigarettes elicit similar biomarker changes to cigarette smoking (10–12) while others found a relative reduction in risk indicators or pre-existing disease after switching from cigarettes to e-cigarettes (13, 14). Nonetheless, to evaluate longer-term health effects, it is essential to identify biomarkers that may be informative of cancer risk related to cigarette and e-cigarette use. Such biomarkers should meet the following criteria to be of clinical use: (i) they should be modified by smoking and e-cigarette use; (ii) they should lie in genes associated with carcinogenesis; (iii) they should indicate a clonal advantage for cells, as indicated by an aggravation in cancer tissue compared with adjacent non-cancer tissue; (iv) they should be associated with cancer progression in a premalignant lesion; and (v) they should be reflective of long-term cancer risk in a surrogate tissue, for example, blood or buccal swab, to allow for noninvasive monitoring.

Investigating how tobacco use or e-cigarettes influence the epigenome, and might thereby be linked to carcinogenesis, could help to better understand their long-term impacts. DNA methylation (DNAme) at the cytosine C-5 position is an epigenetic modification that integrates the impact of heritable and nonheritable factors (15). It has previously been implicated in conveying, at least in part, the long-term health impacts of smoking, with DNAme alterations enriched in genes associated with smoking-related diseases (16). Certain epigenetic changes have shown persistence after smoking cessation (17) and could even predict lung cancer incidence (e.g., methylation in genes *AHRR* or *F2RL3*; refs. 18–20). Investigations into smokeless tobacco (21, 22) or e-cigarette use (22, 23) on DNAme are also emerging. These studies generally report less pronounced epigenetic changes when comparing smokeless tobacco with combustible cigarettes (21, 22), as well as an absence of a strong DNAme response to e-cigarette use in blood (22) and saliva (23).

The majority of DNAme studies into smoking-related changes, including those predicting lung cancer incidence (18, 19), have used blood samples (e.g., refs. 24–29). However, DNAme variations across cell types (30), in particular in response to exposures and other nonheritable factors, merit consideration. For instance, aging has been found to impact DNAme differently across distinct cell types or tissues (31–33). Such findings necessitate the consideration of cellular heterogeneity during DNAme analysis, which is typically carried out in bulk, for the interpretation of epigenetic changes (34, 35). Although many studies in blood have accounted for cellular composition, studies that explore methylation changes in specific cell types remain sparse (36, 37). These studies identified that smoking differentially impacts on cell types of the innate and adaptive immune system (36, 37). Some studies have also investigated DNAme changes in response to smoking in other sample types, including buccal swabs (21, 38, 39), saliva (40), adipose, or skin tissue (41).

Notably, while investigating different tissues or accounting for cellular heterogeneity, few studies have aimed to study the effects of tobacco or e-cigarette use on DNAme across distinct cell types (36, 37), and none have directly scrutinized impacts on epithelial versus immune cells at different anatomic sites (directly exposed vs. not directly exposed). This is of particular interest given the role of epithelial cells, whether directly exposed (e.g., lung, oral mucosa) or not (e.g., cervix), as the predominant cell of origin for tobacco-related malignancies, and the fact that smoking-related DNAme changes in buccal samples, consisting predominantly of epithelial cells, were found to reflect cancer-associated changes (38). Meanwhile, immune cells and their dysregulation can promote tumor initiation and progression (42), and their specific changes in response to smoking might likewise be of relevance.

Investigating cell type–specific DNAme changes resulting from smoking or vaping could therefore help to (i) unveil diverse biological responses to tobacco use by distinct cell types, (ii) identify common or divergent epigenetic alterations elicited by tobacco or e-cigarette use in distinct cell types that might be obscured by bulk analysis, (iii) provide insights into carcinogenesis and potential diagnostic markers. In this study, we systematically unravel the impact of tobacco use on epithelial versus immune cells, employing deconvolution and cell type–specific DNAme inference using data from 1,164 buccal/saliva, 1,777 cervical, and 616 blood samples. We comprehensively assess and validate effects on directly or not directly exposed, thereafter termed “proximal” and “distal”, epithelial and immune cells, in response to smoking, smokeless tobacco, or e-cigarette use. Thereafter, we extend our enquiry into lung cancer tissue and prognosis, along with surrogate samples preceding lung cancer diagnosis to investigate whether smoking-related changes might be suitable for cancer prediction in smokers.

## Materials and Methods

### Study and sample overview

An overview of characteristics of participants and samples is shown in Supplementary Table S1.

#### Discovery set

Buccal, cervical, and blood samples were obtained from healthy volunteers who took part in the FORECEE study (female cancer prediction using cervical omics to individualize screening and prevention—4C), a multicenter study involving several recruitment sites in five European countries (the United Kingdom, Czech Republic, Italy, Norway, and Germany). The FORECEE study had ethical approval from the UK Health Research Authority (REC 14/LO/1633) and all other contributing centers. Participants were ages >18 years and <86 years. After providing written informed consent, participants completed an epidemiologic questionnaire.

Samples were processed as described previously (43). Briefly, buccal cells were collected using two Copan 4N6FLOQ Buccal Swabs (Copan Medical Diagnostics, catalog no. 4504C) by firmly brushing the swab head five to six times against the buccal mucosa of each cheek. The swabs were recapped and left to dry out at room temperature within the sampling tube, which contained a drying desiccant. The sample vial was sealed and stored locally at room temperature. For blood samples, 2.5 mL of venous whole blood was collected in PAX gene blood DNA tubes (BD Biosciences #761165) and stored locally at −20°C. Cervical liquid-based cytology samples were collected at appropriate clinical venues by trained staff using the ThinPrep system (Hologic Inc., catalog no. 70098-002). Cervical cells were sampled from the cervix using a cervix brush (Rovers Medical Devices, catalog no. 70671-001), which was rotated five times through 360 degrees while in contact with the cervix to maximize cell sampling. The brush was removed from the vagina and immersed in a ThinPrep vial containing Preserve-cyt fluid and then pushed against the bottom of the vial 10 times to facilitate release of the cells from the brush into the solution. All samples were shipped to University College London (UCL) at ambient temperature. Biological samples were given an anonymous Participant ID Number, which was assigned to the person's name in a securely stored link file.

Cervical, buccal, and breast tissue DNA were normalized to 25 ng/μL and 500 ng total DNA were bisulfite modified using the EZ-96 DNA Methylation-Lightning kit (Zymo Research Corp, catalog no. D5047) on the Hamilton Star Liquid handling platform. A total of 8 μL of modified DNA was subjected to methylation analysis on the Illumina Infinium HumanMethylationEPIC BeadChip (Illumina) at UCL Genomics according to the manufacturer's standard protocol.

#### Validation set

The validation set comprised 304 matched buccal and blood samples from 152 female volunteers in the UK Medical Research Council (MRC) National Survey of Health and Development (NSHD), a birth cohort study of men and women born in 1946, as described previously (38, 44), and 442 cervical samples from breast cancer cases collected as part of the FORECEE study (see Discovery set). All volunteers in the NSHD study provided written informed consent for their samples to be used in genetic studies of health, and the Central Manchester Ethics Committee approved the use of these samples for epigenetic studies of health in 2012. Women were selected from those who provided a buccal and blood sample at age 53 years in 1999, who had not previously developed any cancer, and who had complete information on epidemiologic variables of interest and follow-up. Methylation analysis for buccal and blood samples was performed using the Illumina Infinium HumanMethylation450 BeadChip array (38), while it was performed using the Illumina Infinium HumanMethylationEPIC BeadChip (Illumina) at UCL Genomics according to the manufacturer's standard protocol in the cervical samples.

#### E-cigarette set

Data on e-cigarette users were derived from the Studying the Epigenetics of E-cigarette Use (SEE-Cigs) study (23). As described previously, e-cigarette users, tobacco smoker, and nonsmokers ages 16 to 35 years were recruited from the UK general population via several mechanisms, including flyers, blogs, podcasts, and social media from January 2017 to January 2019. E-cigarette users were defined as having used e-cigarettes at least weekly for the past 6 months and having smoked less than 100 cigarettes in their lifetime; smokers were defined as having smoked cigarettes at least weekly for the past 6 months and having used an e-cigarette less than 100 times in their lifetime; never smokers were defined as having smoked cigarettes or e-cigarettes less than 100 times in their lifetime. Additional eligibility criteria were good self-reported physical and mental health and ability to give informed consent as judged by the investigator. Exclusion criteria were dependence on alcohol or drugs other than nicotine; significant current or past illness, current pregnancy or breast feeding; having a related individual in the study (23).

After completing an online questionnaire, participants were screened for eligibility and sent an information sheet and consent form. Written informed consent was obtained from all participants. Participants received a saliva collection kit (DNA Genotek Oragene) and were asked to provide 2 mL of saliva. DNA was extracted from saliva samples and underwent bisulphite conversion using the Zymo EZ DNA Methylation kit (Zymo). Genome-wide methylation status of over 850,000 cytosine-phosphate-guanine sites (CpG) was measured using the Illumina HumanMethylationEPIC array according in three batches with sampling criteria in place to ensure that all three groups were represented in each batch to minimize potential confounding by batch effects. Microarray data underwent quality control and normalization using meffil, an R package designed for preprocessing of large samples of Illumina Methylation BeadChip microarrays (45). Sample outliers were identified and removed on the basis of sex chromosome methylation, methylation versus unmethylation intensity, control probes, detection *P* values (*N*  =  10 exclusions in total: 4 vapers, 3 smokers, and 3 nonsmokers). Poor-quality CpG sites, SNP/control probes and CpGs on the sex chromosomes were excluded, resulting in 846,244 CpG sites for analysis.

#### Smokeless tobacco use set

Data on saliva samples from snuff tobacco users, smokers, and nonsmokers were obtained from the “Development of Biomarkers of Effect From Chronic Tobacco Usage” study (NCT01923402; ref. 21). Briefly, a cross-sectional study was conducted between June 2010 and January 2011. Adult male subjects ages 35–60 years were enrolled into three cohorts of 40 subjects each, and written informed consent was obtained from all participants. Smokers were defined as exclusive cigarette smokers who self-reported smoking at least 10 cigarettes per day for at least 3 years; moist snuff tobacco users were defined as self-reporting using at least two cans of moist snuff per week for at least 3 years; nonsmokers were individuals who self-reported not to use any tobacco or nicotine-containing products for at least 5 years. Buccal cells were collected following a 2-hour fasting window from food and tobacco. Subjects rinsed their mouth with Scope mouthwash followed by a water rinse and buccal cells were collected. The cell pellet was washed in PBS and used for DNA extraction. DNA extraction and global methylation profiling of 485,577 CpG sites were performed by Expression Analysis, Inc., on Illumina Infinium HumanMethylation450 BeadChip arrays.

#### Lung cancer tissue

Preprocessed and harmonized Illumina HumanMethylation450K array DNAme data from The Cancer Genome Atlas (TCGA) from lung squamous cell carcinoma (LUSC) and lung adenocarcinoma (LUAD) were accessed via TCGAbiolinks, utilizing all available methylation samples in using project codes TCGA-LUAD and TCGA-LUSC (46). Detailed methods are provided in the code repository.

#### Cervical cancer tissue

DNAme data from cervical cancer tissue or matched normal samples were obtained from NCBI Gene Expression Omnibus (GEO; GSE211668; ref. 47).

#### Carcinoma *in situ* progression data

DNAme data from premalignant precursor lesions [carcinoma *in situ* (CIS)] that either recurred or did not recur were obtained from NCBI GEO (GSE108123; ref. 48). Progressive and regressive lung CIS lesions were laser-captured, and their epigenome interrogated using the Illumina Infinium HumanMethylation450 BeadChip. Data were matched to patient characteristics using Supplementary Materials and Methods, Table 1 from a previous publication (49).

#### ESTHER study set

DNAme data were obtained from participants of the ESTHER (Epidemiological Study on the Chances of Cure, Early Detection and Optimized Therapy of Chronic Diseases in the Elderly Population) study, a large ongoing prospective, population-based cohort study conducted in Germany. In brief, 9,940 participants were recruited by their general practitioners during routine health checkups between July 2000 and December 2002 and provided written informed consent for study participation. The participants have been followed up every 2 to 3 years since then. At baseline recruitment and each follow-up, standardized self-administered questionnaires were used to collect information on sociodemographic characteristics, lifestyle, and dietary factors. Blood samples were collected during the examinations and stored at −80°C for later testing. DNAme analyses in this study were based on 1,352 samples from randomly selected individuals (subset IV, total *n* = 1,493), and analyzed using the Illumina MethylationEPIC. Incident cases of cancer during follow-up between 2000 and end of 2018 (17 years of follow-up) were identified through record linkage with the Saarland Cancer Registry. Controls are participants without lung cancer diagnosis until the end of 17 years of follow-up.

#### General information for clinical studies

All studies obtained written informed consent from participants. Studies were conducted in accordance with the Declaration of Helsinki and approved by Institutional Review Boards.

### DNAme data preprocessing

Methylation microarray data in the discovery, validation, moist snuff tobacco user, and CIS datasets were processed through the same standardized pipeline running in R version 4.2.2. Raw data were loaded using the R package minfi, version 1.36.0 (50). Any samples with median methylated and unmethylated intensities <9.5 were removed. Any probes with a detection *P* value >0.01 were regarded as having failed. Any samples with >10% failed probes, and any probes with >10% failure rate were removed from the dataset. Beta values from failed probes (∼0.001% of the dataset) were imputed using the impute.knn function as part of the impute R package, version 1.62.0. Non-CpG probes (2,932), SNP-related probes as identified by Zhou and colleagues (82,108), and chrY probes were removed from the dataset as previously reported (43). An additional 6,102 previously identified probes that followed a trimodal methylation pattern characteristic of an underlying SNP were removed. Background intensity correction and dye bias correction were performed using the minfi single-sample preprocessNoob function. Probe bias correction was performed using the beta mixture quantile normalization (BMIQ) algorithm of the ChAMP package, version 2.18.3 (51).

For the ESTHER study data, raw DNAme data were normalized to internal controls provided by the manufacturer. In data preprocessing, signals of probes with detection *P* value >0.01, >10% missing values, and probes targeting the X and Y chromosomes were excluded.

Cell type proportions were inferred using EpiDISH (epigenetic dissection of intrasample heterogeneity; ref. 52). Epithelial, fibroblast, and immune cell proportions were identified using the centEpiFibIC.m reference matrix. Immune cell subtype proportions were identified using the hierarchical EpiDISH algorithm (hEpiDISH) with the centBloodSubtype.m reference matrix (maxit = 500, RPC = 3, h.CT.idx = 3).

### Analysis of DNAme association with smoking

Our analysis workflow is shown in Supplementary Fig. S1. We evaluated cell type–specific DNAme changes associated with smoking separately in DNAme data buccal, cervical, and blood samples of current or never smokers (Supplementary Table S1). Initially, we conducted an epigenome-wide association study (EWAS) separately in each tissue, accounting for age and immune cell proportion (buccal, cervical samples), or age and lymphoid cell proportion [blood, 1 – (myeloid proportion)], utilizing hEpiDISH (53). We grouped monocytes, neutrophils, and eosinophils as myeloid lineage (hepidish_Mono, hEpidish_Neutro, hEpidish_Eosino).

CpGs were considered significantly associated with smoking if their Holm–Bonferroni–corrected *P* value was < 0.05, corresponding to *P* < 8.2 × 10^−8^ for cervical and *P* < 7.9 × 10^−8^ for buccal and blood samples, which is more conservative than a benchmarking study for the EPIC array suggested (*P* < 9 × 10^−8^; ref. 54). To estimate the impact of smoking on epithelial versus immune cells in buccal and cervical samples, we performed linear regression of the beta values on EpiDISH-inferred immune cell proportion (52) for each CpG site, as described previously (43, 55). The linear models were fitted for smokers and never smokers separately, and the intercept points at immune cell proportion = 0 were used as estimates of mean beta values in smokers and never smokers in a pure epithelial cell population. The difference between these intercept points provided a Δ β estimate in epithelial cells. Conversely, the difference between intercept points at immune cell proportion = 1 provided immune cell Δ β estimates. The same approach was applied to account for myeloid and lymphoid differences.

All CpGs that were (i) significant in at least one of the samples after Holm–Bonferroni correction, (ii) present on Illumina Human MethylationEPIC array version 2, and (iii) not on our list of previously identified “unreliable” probes were used for further analysis (*n* = 535; ref. 56). Of note, seven of these CpGs are located on the X chromosome and were removed for evaluation of mean scores in additional datasets.

We performed clustering on a reduced feature space to identify co-regulated groups of CpGs, that is, a matrix of Δ β values where rows were based on CpGs that were significantly associated with smoking in the initial EWAS, and columns were based on Δ β values of the given CpG across all tissues (Δ β epithelial in buccal, Δ β immune in buccal, Δ β epithelial in cervical, Δ β immune in cervical, Δ β lymphoid in blood, Δ β myeloid in blood), constituting a matrix of 535 × 6 (Supplementary Fig. S1). Clusters were identified via Uniform Manifold Approximation and Projection (UMAP) and validated using a distance-based hierarchical clustering approach.

### Functional annotation and gene set enrichment analysis

The Illumina Infinium HumanMethylationEPIC BeadChip manifest (doi: 10.18129/B9.bioc.IlluminaHumanMethylationEPICanno.ilm10b4.hg19) was used to identify genes the CpGs were spanning. CpGs on sex chromosomes were excluded. The clusterProfiler package (57) was used for gene set enrichment analysis of genes unique to each group (i.e., not present in other groups). All genes with CpGs on the EPIC array not located on sex chromosomes were used as background. Reactome pathway analysis was conducted using ReactomePA package (58) with the *P*valueCutoff set to 0.2 and minGSSize set to 3. *P* values were adjusted using Benjamini–Hochberg method.

Polycomb group target (PCGT) genes were defined genes with occupancy of at least one of SUZ12, EED, and H3K27me in a previous chromatin immunoprecipitation sequencing experiment (Supplementary Table S9 in ref. 59). Of these, 1,343 genes were found in the Illumina Infinium HumanMethylationEPIC manifest. Enrichment for PCGT genes was conducted via Fisher exact test.

### Association with gene expression

Matched gene expression (STAR counts) and methylation data were obtained from TCGA-LUAD and TCGA-LUSC via the TCGABiolinks package. For each CpG, methylation beta values were correlated to log_2_ corresponding *cis* gene counts (Pearson correlation). *P* values and Pearson R were collected and visualized. CpGs with a correlation of Holm–Bonferroni–corrected *P* value < 0.05 were considered significantly associated with gene expression.

### Mean methylation computation and correction for cell type

Mean methylation beta value $\bar{\beta }\ $(mean β) for each set of CpGs was calculated as:
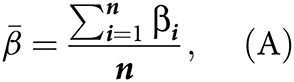
where β_*i*_ represents the beta value of each CpGs and *n* is the total number of CpGs in each set. Datasets derived from the IlluminaMethylationEPIC array would use all sites unless specifically indicated (i.e., when directly comparing 450K and EPIC array), whereas from the 450K array would only use sites present on the 450K array. Performance of mean methylation values did not seem to depend on Illumina Methylation array version, although the 450K array only included approximately half of the relevant smoking site CpGs.

Our epigenome-wide analysis revealed that cell type heterogeneity can influence methylation scores at sites associated with smoking. To account for cell type heterogeneity in buccal or saliva samples and infer methylation values of a “pure” sample consisting either of only epithelial or immune cells, we applied a correction algorithm. Briefly, for each type (never, ex-smokers, current smokers; or e-cigarette users, moist snuff tobacco users), a linear model was fit for mean methylation value against immune cell proportion. For each score ${{\bar{\hskip -2pt\beta }}}$ and type *t*, the residual between true and predicted value was then added to the intercept at immune cell proportion = 0 (“pure” epithelial sample; for epi hypomethylated (hypoM), distal epithelial hypermethylated (hyperM), and proximal epithelial hyperM) or immune cell proportion = 1 (“pure” immune sample; for immune hypoM).
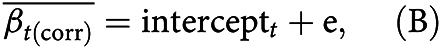
where *t* is type (e.g., never smoker, ex-smoker, current smoker), intercept is the intercept of the model for type *t* at immune cell proportion 0 or 1 (depending on whether an epithelial or immune effect is to be estimated), and e is defined as the residual $y - \hat{y}$ [y = $\bar{\beta }$, i.e., the mean beta value in the set as computed in Eq. A], and $\hat{y}$ is $\widehat {\overline {{{{\mathrm{\beta }}}_t}} }$, that is, the mean estimated value based on the linear regression model in type *t.*

### Statistical analysis

All analyses were conducted in R version 4.3.1. Comparison of mean beta values for between smokers, never smokers, ex-smokers, e-cigarette users, or moist snuff tobacco users, were conducted using Wilcoxon test (paired where indicated). Area under the ROC and corresponding confidence intervals (CI) were computed using the pROC package 1.18.0 (60), utilizing DeLong's method for CI computation. ORs for immune hypoM in the ESTHER study were computed after standardising immune hypoM values, using logistic regression.

### Code availability

Code used in this analysis is deposited under https://github.com/chiaraherzog/WID_SMK_code/.

### Data availability

Data accession numbers for smoking datasets are shown in Supplementary Table S1. Data of the discovery set are deposited in the European Genome-Phenome Archive under study ID EGAS00001005055. Data in the validation set are not deposited because of restrictions on the informed consent of the NSHD cohort but can be requested via https://nshd.mrc.ac.uk/. All proposals to use NSHD data must support and adhere to the core principles of data sharing with the MRC (ethical, equitable, efficient). Data of the e-cigarette set were obtained from the original authors of the SEE-Cigs study. Data on smokeless tobacco use were obtained from NCBI GEO, under accession number GSE94876.

Data on lung cancer were obtained from TCGA. Data on CIS progression were obtained from NCBI GEO under accession number GSE108123. Data on cervical cancer were obtained from NCBI GEO under accession number GSE211668.

All data that support the findings of the ESTHER study are available upon request from the co-author Hermann Brenner. The data are not publicly available due to them containing information that could compromise research participant privacy/consent. All other raw data are available upon request from the corresponding author.

## Results

### Smoking elicits cell type–specific functional epigenetic alterations across epithelial and immune cells depending on anatomic site

**Figure 1.**
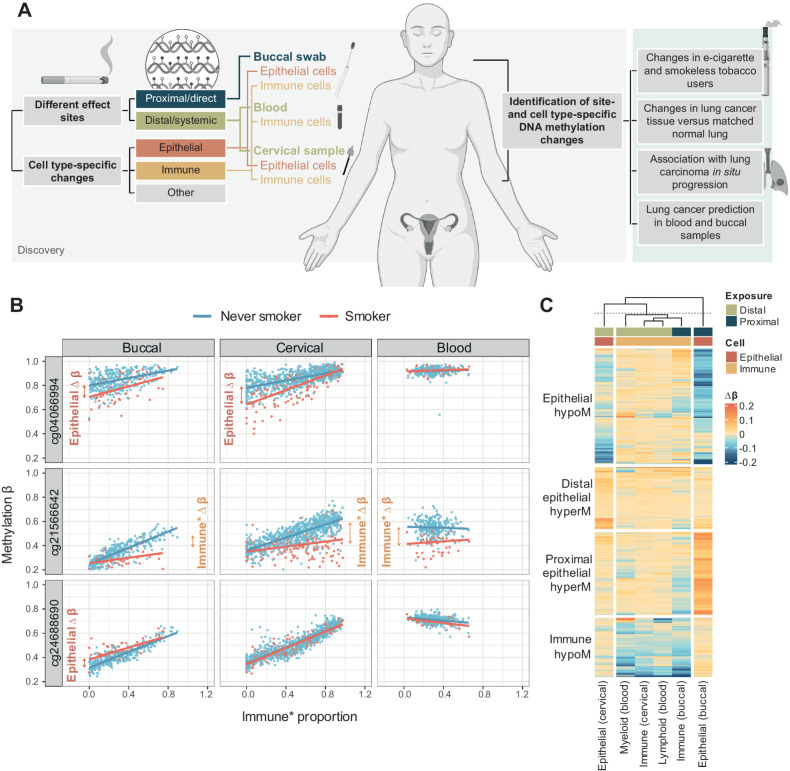
General overview of the study and identification of cell type–specific smoking-dependent epigenetic changes. **A,** Overview of the study. We aimed to identify cell- and tissue-specific epigenetic alterations and used a discovery set of buccal, cervical, and immune cells (all female). Findings were then validated in several independent sets to confirm the association with current and former smoking and explore association of cell-specific effects across smoking alternatives (e-cigarette use, moist tobacco use), lung cancer tissue and progression, and possibility to predict lung cancers in smokers using noninvasive samples. A detailed workflow of the analysis is shown in Supplementary Fig. S1. **B,** Scatterplots of methylation beta values in three CpGs located in the *AHRR* gene or intergenic region versus immune cell proportion (buccal and cervical samples) or lymphoid proportion (blood) indicate methylation differences may be derived from distinct cell types. **C,** Visualization of delta-beta values across four groups of CpGs identified in Supplementary Fig. S5A. A matrix of inferred delta-beta values across all tissues for all significant CpGs (i.e., significant in at least one tissue in the EWAS) was clustered using UMAP and the following clusters identified: epithelial hypomethylation (epithelial hypoM), immune hypomethylation (immune hypoM), distal epithelial hypermethylation (distal epithelial hyperM; effects in distal epithelium but not directly exposed epithelium), and proximal epithelial hypermethylation (proximal epithelial hypoM; effects in buccal/directly exposed samples only). (**A,** Created with BioRender.com.)

Our analysis workflow is shown in Supplementary Fig. S1. Initially, to identify DNAme changes across diverse tissues that are either directly exposed or not directly exposed to tobacco (Fig. 1A), we conducted an EWAS of DNAme levels and smoking status in a discovery set of 542 buccal, 464 blood samples, and 1,335 cervical samples from current or never smokers, including samples from women as these enabled access to both directly exposed and indirectly exposed epithelium (cervix). Characteristics of the discovery set participants are shown in Supplementary Table S1.

The EWAS was conducted separately per sample type, accounting for age and cell type proportion. As expected on the basis of previous reports, we identified multiple CpG loci significantly associated with smoking in buccal and blood samples, and additionally for the first time describe loci associated with smoking in cervical samples (Supplementary Fig. S2–S4a: Manhattan plots; b, quantile-quantile plots; c, delta-beta histogram in buccal, blood, and cervical samples, respectively). We report a total of 535 sites significantly associated with smoking in at least one of the tissues, 279 (52%) of which are also present on the IlluminaHumanMethylation450K (Supplementary Table S2).

To investigate cell lineage–specific effects, we were additionally interested in whether the signal within each tissue was derived from epithelial or immune cells (buccal/cervical) or myeloid or lymphoid cells (blood). To investigate this, we fitted linear models for smokers and never smokers versus immune cell proportion within each sample type and inferred the difference in methylation levels, termed delta beta (Δ β), in pure epithelial or immune cells for buccal or cervical samples [see Materials and Methods and Supplementary Fig. S1, as described previously (43, 55)]. For blood, the same approach was applied but the term immune cell proportion was replaced with lymphoid proportion, based on (1 − inferred sum of monocyte, neutrophil, and eosinophil proportion; Materials and Methods). Among the 535 sites significantly associated with smoking in the EWAS after Bonferroni correction, we identified several loci that exhibited lineage-specific methylation changes. In Fig. 1B, we specifically visualize three example CpGs, located within the *AHRR* gene or intergenic region, that appear to exhibit distinct methylation changes depending on tissue and cell type: for instance, cg04066994 exhibits more pronounced hypomethylation with decreasing immune cell proportion in smokers compared with nonsmokers (i.e., “epithelial differential methylation”), while the hypomethylation is not evident in blood samples or cervical samples with higher immune cell proportions. cg21566642 shows the opposite behavior, indicating differential methylation is driven by immune cells. Moreover, differential methylation of cg21566642 in smokers seemed more pronounced in samples with a lower lymphoid proportion, suggesting a stronger differential methylation in cells of the myeloid lineage. cg24688690 shows differential methylation in buccal but not cervical epithelial cells of smokers compared with never smokers, suggesting methylation changes may be observed only in epithelial cells that are directly exposed to smoke. These examples highlight that even within the same gene, differential methylation signals may be derived from different cell types.

To more systematically classify the 535 significant loci listed in Supplementary Table S2, we conducted data-driven clustering on a reduced feature space, whereby we clustered CpGs based on a matrix of their Δ β values in each sample and inferred cell type (Supplementary Fig. S1) via UMAP. Our approach proposed the existence for four distinct groups of CpGs (Supplementary Fig. S5A), which was also confirmed by an independent distance-based hierarchical clustering approach (Supplementary Fig. S5B). Visualization of Δβ values by cluster indicated that groups were, as expected, largely driven by cell type specificity (Fig. 1C). For simplicity, groups were subsequently named after their predominant pattern: (general) epithelial hypoM CpGs, hypomethylated in both proximal and distal epithelial (buccal and cervical) but not immune cells; immune hypoM CpGs, showing a loss of methylation across all immune samples but not epithelial cells; distal epithelial hyperM CpGs, hypermethylated in distant epithelial cells with few other changes; and proximal epithelial hyperM CpGs, which showed hypermethylation in buccal epithelial cells (Fig. 1C).

**Figure 2.**
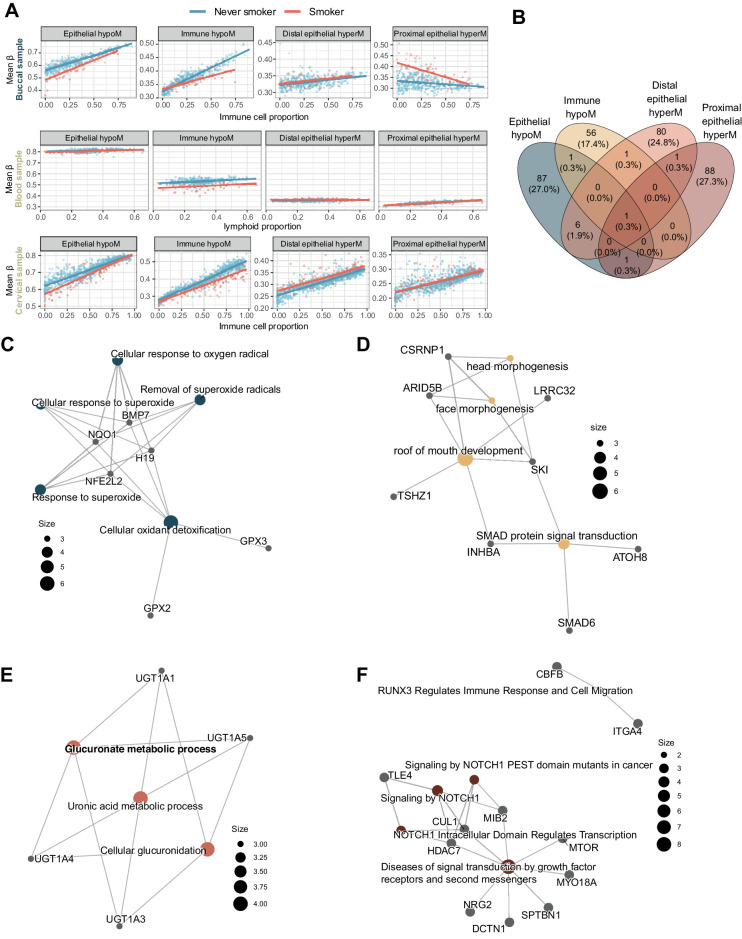
Combined methylation scores of CpGs in the four sets and annotation. **A,** Association of mean methylation β values in each of the sets described in Fig. 1C with immune cell proportion in buccal and cervical samples and lymphoid proportion in blood samples in the discovery set. **B,** Venn Diagram of genes associated with CpGs in each of the four smoking-associated sets of CpG indicates little overlap between involved genes. **C–F,** Gene ontology (**C–E**) and Reactome pathway enrichment (**F**) for the four sets of smoking-associated CpGs reveals different pathways.

Figure 2A illustrates the mean β value across all CpGs in each of the four groups against immune cell proportion (buccal and cervical sample) or lymphoid proportion (blood) in the discovery set, and confirmed cell type–specific effects: for example, epithelial hypoM exhibited a loss of methylation with decreasing immune cell content in both buccal and cervical samples, but no difference in blood samples, indicating a general epithelial effect, whereas proximal epithelial hyperM specifically emerged with decreasing immune cell content in buccal samples, but not in cervical (or blood) samples.

Aiming to investigate whether the four groups of CpGs were associated with specific genes or functions, we found CpGs in the four groups shared little overlap in the genes that they were spanning (Fig. 2B), with only one gene being shared between all three groups (*AHRR*). Gene set enrichment identified specific pathways for each group (Fig. 2C–F; Supplementary Tables S3–S6): genes unique to epithelial hypoM CpGs were enriched for involvement in cellular response to oxidative stress and detoxification, immune hypoM CpGs were uniquely associated with genes involved in morphogenesis and development; distal epithelial hyperM CpGs were uniquely associated with genes involved in glucoronate and uronic acid metabolism, and Reactome pathway analysis revealed that proximal epithelial hyperM CpGs were associated with genes involved in *NOTCH1*/*RUNX3*/growth factor receptor signaling and transduction, and included genes *HDAC7* and *MTOR*. Proximal epithelial hyperM and immune hypoM sites exhibited an enrichment for genes covered by PCGTs, that are known regulators of cell fate (Supplementary Fig. S6A).

Leveraging matched expression and methylation data for CpGs present on the 450K array in lung tissue derived from TCGA, we assessed whether individual CpGs were associated with expression of *cis* genes. This indicated that several methylation loci were significantly associated with expression, and 55/98 loci with matching expression data exhibited *P* < 0.05 after Bonferroni correction (Supplementary Fig. S6B; Supplementary Table S7). Depending on their regulatory position, CpG loci that were significantly correlated with expression after Bonferroni correction were associated with negative (transcription start site) or positive regulation of expression (body; Supplementary Fig. S6C).

We investigated how many of the sites overlapped with those identified by previous studies. Except for immune hypoM CpGs, the majority of CpGs (320/535, 60%) were not previously reported, likely due to the fact that the majority of prior studies utilized blood samples containing immune cells only (Supplementary Fig. S6D; refs. 16, 28, 38).

As female-only samples were used for discovery, seven of 535 CpGs were on the X chromosome and were excluded from further analyses, some of which also contained samples from male donors, resulting in 528 CpGs evaluated the remainder of the study.

### Smoking-related cell-type specific effects are attenuated in former smokers

**Figure 3.**
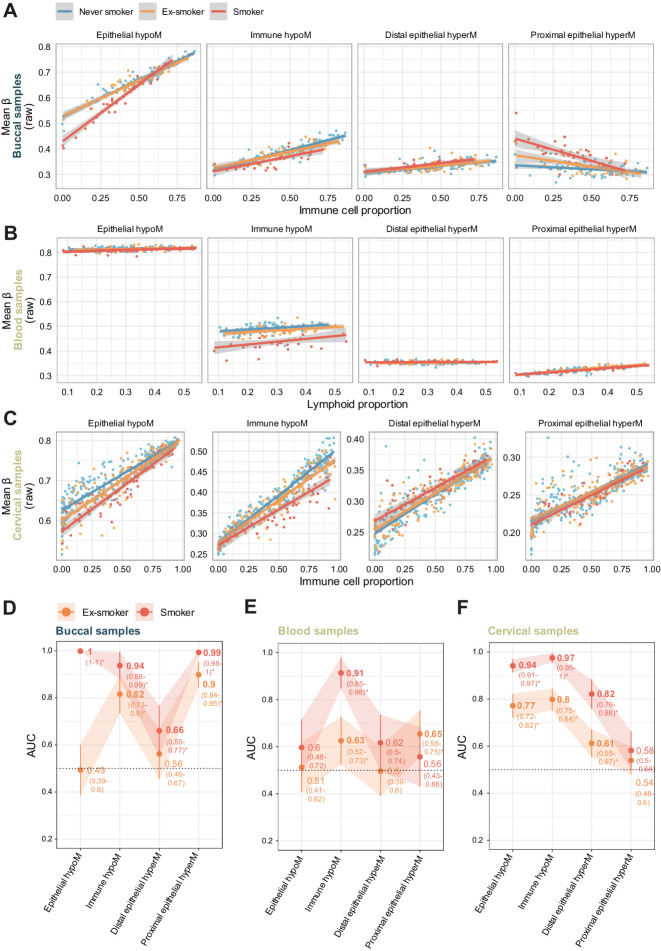
Evaluation of scores in independent validation sets. Independent dataset comprising 304 matched blood and buccal samples (*n* = 152 each) and 442 cervical samples was used to validate the findings. **A–C,** Mean beta values (uncorrected) in each of the four sets of CpGs in buccal (**A**), blood (**B**), and cervical (**C**) samples of never smokers, ex-smokers, and current smokers versus immune cell proportion (**A** and **C**) or lymphoid proportion (**B**). **D–F,** AUC of corrected values in each of the four sets of CpG comparing never smokers with current or former smokers in buccal (**D**), blood (**E**), and cervical (**F**) samples. Mean methylation scores in this figure only include sites present on the 450K array for comparability between datasets.

We initially validated our findings in a dataset of 152 matched buccal and blood samples (450K array), as well as a separate set of 442 cervical samples (EPIC array), derived from never smokers, ex-smokers, or current smokers (Fig. 3A–C) by visualizing mean methylation in each group versus inferred immune or lymphoid cell proportion, which revealed groups of CpGs behaved similarly as in the discovery set (Fig. 2A).

To enable the comparison of each group of CpGs to distinguish between never smokers, ex-smokers, or current smokers using the area under the ROC curve despite differences in cellular composition (cell type distributions across all datasets in this study are shown in Supplementary Fig. S7A and S7B), we applied a correction algorithm, illustrated in Supplementary Figs. S1, S8a–S8d, and in Supplementary Data S1. Similar to the initial discovery approach to infer delta-betas in pure epithelial or immune cell proportions, this correction allowed us to estimate the methylation level in a pure epithelial or immune cell fraction derived from a given sample. Corrected mean beta values in each group of CpGs showed AUC values in line with what would be expected (Fig. 3D–F): for example, in blood the immune hypoM score performed best whereas the mean methylation of epithelial-derived CpGs did not result in a high AUC (Fig. 3E), indicative that epithelial-specific differential methylation does not distinguish smokers from never smokers in immune samples. Epithelial hypoM signature distinguished smokers from never smokers in both cervical and buccal samples whereas the proximal and distal epithelial hyperM signatures exhibited a high ability to distinguish smokers from never smokers only in relevant proximal (buccal) or distal (cervical) samples containing epithelial cells, respectively (Fig. 3D–F).

As reported previously, the methylation changes in former smokers were less pronounced than in current smokers, in relation to never smokers. In buccal samples, the mean corrected beta value of epithelial hypoM CpGs was not significantly different from never smokers, whereas the same signature remained differentially methylated in cervical samples of ex-smokers (Fig. 3D–F). Proximal epithelial hyperM also remained significantly elevated in buccal samples from former smokers compared with never smokers (Fig. 3D). Across all samples, the immune hypoM signature was significantly differentially methylated in never smokers compared with controls (Fig. 3D–F).

To study dose dependence of smoking signatures, we investigated their association with smoking pack year in buccal and blood samples, for which we had this information available. Smoking pack years were significantly correlated with the mean methylation levels of relevant groups of CpGs in each tissue (immune hypoM in blood, all except distal epithelial hyperM in buccal samples; Supplementary Fig. S9A and S9B).

### E-cigarette and smokeless tobacco use alter the epigenome of oral epithelial cells similar to cigarette smoking

**Figure 4.**
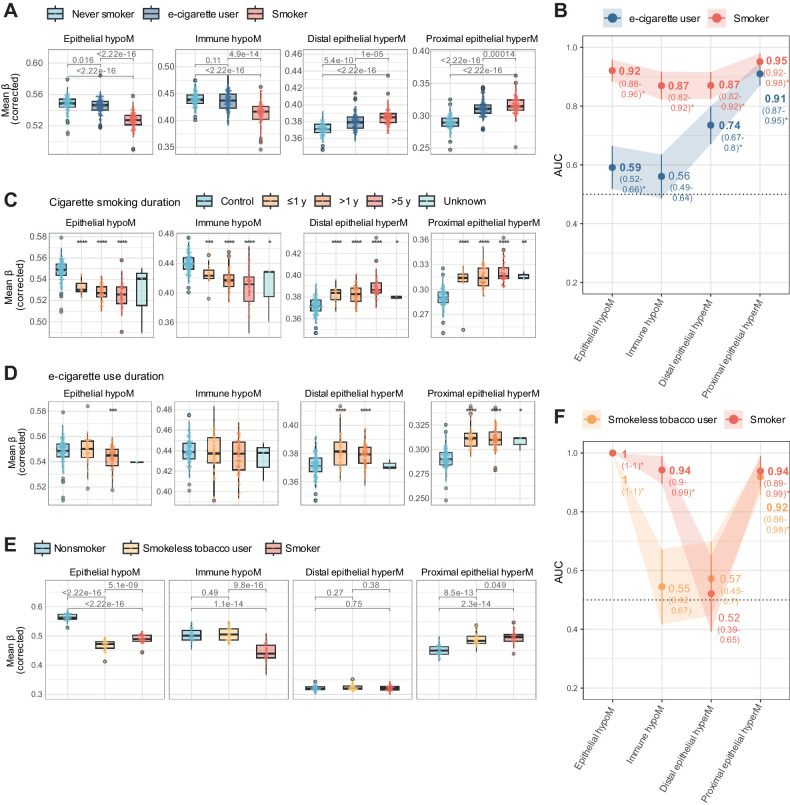
Impact of e-cigarette and smokeless use on cell type–specific epigenetic smoking signatures. **A,** Mean beta values (corrected) in each of the four sets in saliva samples of never or current smokers or e-cigarette users, corrected for cell type–specific effects. **B,** AUC of corrected values in each of the four sets comparing smokers or e-cigarette users with controls in the e-cigarette use dataset. **C,** Mean beta values in each of the four sets in never smokers (control) or smokers, stratified by categorial smoking duration information. **D,** Mean beta values in each of the four sets in never smokers (control) or e-cigarette users, stratified by categorial e-cigarette duration information. The legend is identical to **C**. **E,** Mean beta values (corrected) in each of the four sets in saliva samples of current nonsmokers (prior smoking history not known), smokeless tobacco users, or smokers in the smokeless tobacco use set. **F,** AUC of corrected values in each of the four sets of CpGs comparing nonsmokers with smokeless tobacco users or smokers in the smokeless tobacco use set. *, *P* < 0.05; **, *P* < 0.01; ***, *P* < 0.001; ****, *P* < 0.0001 in Wilcoxon test compared with relevant controls (never or nonsmokers, respectively, for **A**, **C**, **D**, and **E**).

We next evaluated corrected methylation scores in saliva samples from never smokers, e-cigarette users who smoked less than 100 cigarettes in their life, and current cigarette smokers (Fig. 4A–D; raw and corrected values in Supplementary Fig. S10A and S10B). Whereas e-cigarette users did not have significantly different levels of immune hypoM levels from controls, they exhibited altered levels of proximal epithelial hyperM [AUC: 0.91 (95% CI: 0.87–0.95)], distal epithelial hyperM CpGs [AUC: 0.74 (0.67–0.80)], and epithelial hypoM [AUC: 0.59 (0.52–0.66)] compared with controls (Fig. 4A and B). E-cigarette users had a limited smoking history (<100 cigarettes in their life), and methylation levels were not correlated with reported smoking history, except for immune hypoM (Supplementary Fig. S10C), or mL of e-cigarette liquid used per day as a quantitative proxy for e-cigarette use frequency (Supplementary Fig. S10D). Categorical information was available on duration of smoking or e-cigarette use, respectively (≤1 year, > 1 year, > 5 years). In smokers, the smoking-related changes became more pronounced with increasing duration for epithelial hypoM and immune hypoM but were less time dependent for proximal or distal epithelial hyperM (Fig. 4C). Likewise, proximal and distal epithelial hyperM changes in e-cigarette users appeared sooner (<1 year) than epithelial hypoM (≤1 year), the latter of which was significantly different from controls only in after 1 year or more of reported vaping (Fig. 4D).

To better understand the similarities and differences of smoking and e-cigarette use, we next assessed the inferred epithelial and immune delta beta value at the individual 528 CpG sites. This revealed a partial but not complete overlap between smokers in the discovery set and e-cigarette users (Supplementary Fig. S11A). Sites overlapping for proximal epithelial hyperM in the inferred epithelial fraction were still enriched for sites associated with growth factors and damage response and notably included genes such as *HDAC7* and *MTOR* (Supplementary Fig. S11B), while epithelial hypoM sites remained enriched for cellular response to chemical stress, including genes such as *NFE2L2* and *GPX2/3* (Supplementary Fig. S11C; Supplementary Tables S8 and S9).

To compare cigarette and e-cigarette use to smokeless tobacco, we next evaluated methylation changes in moist snuff users. Saliva samples from smokeless tobacco users exhibited significant differences in epithelial hypoM and proximal epithelial hyperM, but not immune hypoM, compared with nonsmokers (Fig. 4E) and these signatures were highly discriminative between nonsmokers and smokeless users (Fig. 4F; AUCs of 1 and 0.92, respectively; raw values for mean beta in each group of CpGs are shown in in Supplementary Fig. S12A and S12B).

### Smoking-associated methylation alterations are associated with cancer and CIS progression

Smoking-associated changes in buccal cells were previously found to be associated with cancer-related changes (38). We were therefore interested in whether one or more of the four sets of functionally distinct CpGs showed a particular association with current or future cancers associated with smoking.

**Figure 5.**
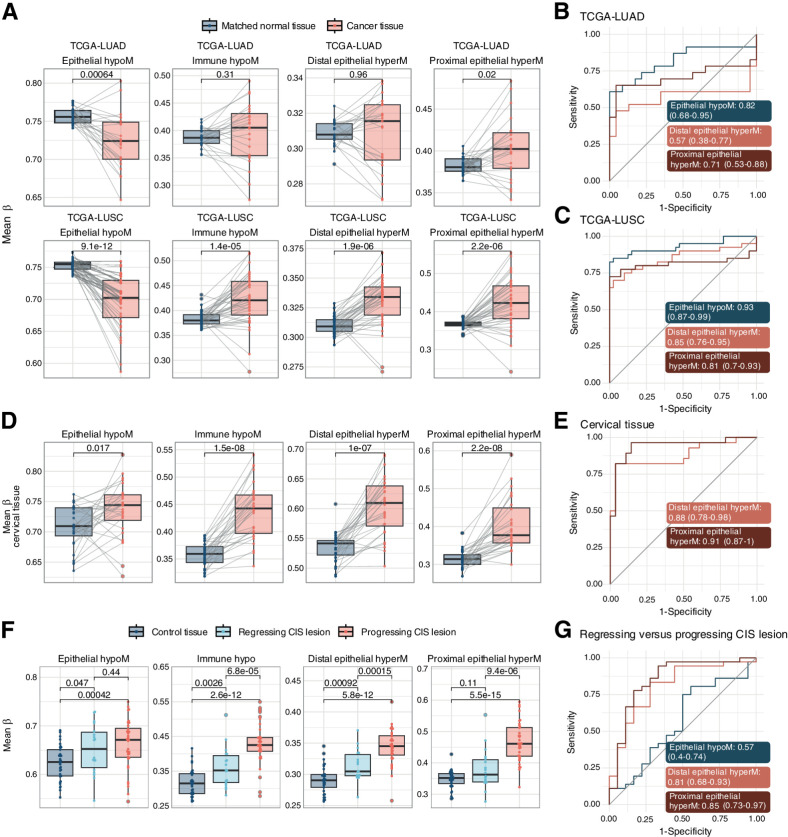
Mean methylation beta of smoking-associated CpG sets in cancer tissue and progressing versus regressing CIS lesions. **A,** Mean methylation beta values in each set in TCGA LUAD and LUSC projects. Only samples with matched normal control tissue were included to control for smoking exposure. *P* values are derived from a paired Wilcoxon test. **B** and **C,** AUC plots for mean methylation levels in epithelial hypoM, distal epithelial hyperM, and proximal epithelial hyperM, comparing matched control tissue versus lung cancer tissue in TCGA-LUAD (**B**) and TCGA-LUSC (**C**). **D,** Mean methylation beta values in each set in cervical cancer or matched normal tissue (GSE211668). Only samples with matched normal control tissue were included to control for smoking exposure. *P* values are derived from a paired Wilcoxon test. **E,** AUC plots for mean methylation levels in epithelial hypoM, distal epithelial hyperM, and proximal epithelial hyperM, comparing matched control tissue versus cervical cancer tissue (GSE211668). **F,** Mean methylation beta values in the smoking-associated CpG sets in control lung tissue, regressing CIS lesions, or progressing CIS lesions. *P* values are derived from paired Wilcoxon tests. **G,** AUC plots for mean methylation levels in epithelial hypoM, distal epithelial hyperM, and proximal epithelial hyperM, comparing matched regressing CIS versus progressing CIS lesions.

Mean methylation levels of the each of the four sets of CpGs in lung cancer samples from LUAD or LUSC in TCGA revealed similar changes compared with smoking for epithelial hypoM, distal epithelial hyperM, and proximal epithelial hyperM when compared with matched normal tissue to control for smoking exposure (Fig. 5A–C). For instance, proximal epithelial hyperM CpGs exhibited consistent hypermethylation in the tumor compared with matched normal tissue, whereas epithelial hypoM showed consistent hypomethylation. Sets that showed opposing directions between cancer tissue compared with smoking were excluded from AUC graphs in Fig. 5 (e.g., immune hypoM CpGs; Fig. 5B and C).

Cigarette smoking is also associated with cancers at non-directly exposed sites, including cervical cancer, and we hypothesized that smoking-related CpGs might be associated with these cancers as well. Distal epithelial hyperM CpGs, identified in cervical samples, were significantly hypermethylated in cervical cancer tissue. Interestingly, also proximal epithelial hyperM was significantly hyperM in cervical cancer tissue, possibly due to its role in cancer-related genes (Fig. 5D and E).

As established cancers often exhibit a highly disrupted epigenome and might therefore not be as informative regarding early alterations driving cancer progression, we also investigated mean methylation levels of each of the four sets of CpGs in CIS lesions, that can either progress to cancer or regress. In particular, the proximal epithelial hyperM was highly elevated in CIS lesions that later progressed to cancer, while it was not significantly elevated in regressing lesions (Fig. 5F). Proximal epithelial hyperM distinguished between progressing and regressing lesions with an AUC of 0.85 (0.73–0.97; Fig. 5G). Dependence of mean methylation values on immune cell proportion for lung tissue, cervical tissue, and CIS samples is shown in Supplementary Fig. S13A–S13C.

### Prediction of lung cancer using blood and buccal samples

Previous studies have indicated lung cancer may be predicted via methylation levels in blood samples, which could help with risk stratification for screening methodologies such as low-dose CT. We were interested in comparing the immune-related set of CpGs discovered in the current study to previous predictors. Moreover, as some of the sets were associated with cancer or CIS progression in lung tissue, we wondered whether they might be able to distinguish between future cancer cases in controls on buccal samples from current smokers.

**Figure 6.**
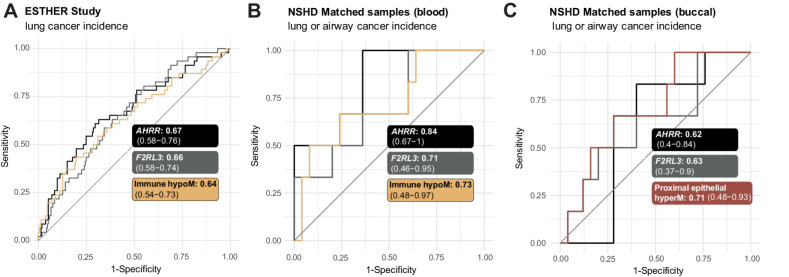
Prediction of lung cancer using immune hypoM in blood and proximal epithelial hyperM in buccal samples compared with previously described predictors. **A,** Comparison of the AUCs of *AHRR* (cg05575921), *F2RL3* (cg03636183), and mean methylation at immune hypoM to identify any lung cancer cases within 17 years in 259 current smokers in the ESTHER study. **B,** Comparison of the AUCs of *AHRR* (cg05575921), *F2RL3* (cg03636183), and mean methylation at immune hypoM (corrected for immune cell proportion) to identify any lung or airway cancer cases within 22 years in 31 blood samples (*n* = 6 cancer cases) of the validation set (same individuals as in **C**). **C,** Comparison of the AUCs of *AHRR* (cg05575921), *F2RL3* (cg03636183), and mean methylation at proximal epithelial hyperM (corrected for immune cell proportion) to identify any lung or airway cancer cases within 22 years in 31 buccal samples (*n* = 6 cancer cases) of the validation set (same individuals as in **B**).

Assessment of immune hypoM signature in 1,352 blood samples derived from the ESTHER study with complete smoking pack-year information (Supplementary Table S10), including samples from controls and cases who developed lung cancer up to 16.8 years after sample donation, indicated that one SD increase in immune hypoM was associated with significantly reduced OR of developing lung cancer [OR = 0.96 (95% CI: 0.94–0.97), *P* = 1.64e-07] (Supplementary Table S11). However, the effect was modest and comparing AUC indicated that no significant gain in information could be achieved in comparison with previously identified single-site predictors *AHRR* or *F2RL3* (Fig. 6A).

Follow-up information on lung or airway cancer incidence in the 22 years following sample collection was available for the validation set of matched blood and buccal samples. While sample numbers were small (*n* = 31, 6 cancer cases), *AHRR* alone had the highest AUC in blood (Fig. 6B). Conversely, in buccal samples, proximal epithelial hyperM exhibited the highest AUC (0.71; Fig. 6C).

## Discussion

Several previous studies have investigated smoking-induced DNAme alterations, primarily conducted in blood (16, 28). Recent studies have highlighted the importance of accounting for cell type heterogeneity when investigating DNAme, including cell lineage, when evaluating impacts of smoking in blood (36). Our data provide a first insight into cell type–specific and tissue-specific epigenetic alterations in response to smoking as an external exposure across various cell types and tissues, looking primarily at epithelial versus immune cells, by applying deconvolution and linear models. Importantly, this approach enabled investigation of cell type–specific alterations that are shared by cigarette smoking and e-cigarette use, may be associated with carcinogenesis, and could form the basis for novel cancer detection or risk stratification approaches using self-collected buccal or saliva samples pending additional optimization and validation.

Goldfarbmuren and colleagues recently showed that smoking can induce both pan- and cell-specific changes using single-cell RNA sequencing of the airway epithelium of smokers and nonsmokers (61). Their findings indicated that smoking also induces changes in “protected” stem and submucosal gland cells. In absence of large-scale single-cell methylation datasets from various tissues with regards to smoking, we employed a cell type deconvolution-based approach. Although obtained via a different modality and investigating different cell types, our data are in line with these findings: on one hand, we identify general epithelial effects elicited by cigarette smoking (epithelial hypoM). These DNAme changes occur both in directly exposed and not directly exposed cell types, while on the other hand, we identify DNAme alterations specific to certain cell types and contexts, for example, changes occurring in directly exposed epithelial cells (proximal epithelial hyperM) or not directly exposed epithelial cells (distal epithelial hyperM; Fig. 2A). In line with another recent study (36), our data indicate that effects of smoking for some sites more pronounced in the myeloid than lymphoid lineage (Fig. 1B). Importantly, the total of 535 sites, grouped into four sets of CpGs, shared little overlap in the genes they spanned (Fig. 1E) and were associated with distinct functions. For instance, epithelial hypoM sites were associated with detoxification responses (Fig. 2C), whereas proximal epithelial hyperM sites were associated with growth signaling and DNA damage responses (Fig. 2F). In addition, our findings indicate that methylation levels at CpGs identified in this study were significantly associated gene expression at *cis* genes (Supplementary Fig. S6B and S6C). A limitation of the current study is that we employed pathway analysis based on gene names and limited our investigation to *cis* genes. Future studies will be required investigate the link between methylation changes and gene transcription and function in more detail, including via multiomics profiling (e.g., methylation and gene transcription) of bulk sorted or single cells in various tissues.

Previous studies have investigated epigenetic changes and their reversal in current and former smokers (16, 17, 62). In line with these studies, our data indicate a partial reversibility of smoking-induced epigenetic alterations in former smokers (Fig. 3). For instance, epithelial hypoM, a signature associated with detoxification, was unable to distinguish ex-smokers from never smokers in our buccal sample validation set while it was highly elevated in current smokers (Fig. 3D). We note that to date neither the precise mechanisms of DNAme induction (or loss) upon smoking nor the kinetics and causes of reversal are known. If smoking induces DNAme hypermethylation at a site and changes persist after giving up smoking, it could imply that (i) either the individual cell survived or (ii) the site was methylated in a stem cell and is propagated. Conversely, if the hypermethylation disappears after smoking cessation, it could imply that either (i) the cell has died and been replaced by another cell or (ii) that the smoking-associated methyl group has been actively displaced in a living cell. Methylation patterns may also be influenced by tissue-dependent cell turnover rates (that, in turn, may be affected by smoking), and tobacco “dose”; for instance, relatively longer-lived cells (e.g., lymphocytes) may have more chance to accumulate methylation changes than shorter-lived cells (e.g., neutrophils). Changes in DNAme upon smoking and its cessation may therefore be the result of a combination of cell-specific enzymatic activity related to methylation/demethylation, cell turnover, stem cell involvement, and dose differences. While studies investigating DNA mutation suggest that quitting smoking drives gradual replenishment of bronchial epithelium from cells that have avoided tobacco mutagenesis (63), suggesting at least in part that some stem cells may escape tobacco-related changes, other findings indicate that smoking can also induce gene expression changes in stem cells (26, 61). Longitudinal sample sets (e.g., as collected in ref. 62 and ClinicalTrials.gov NCT05678426), possibly in combination with single cell and tracing experiments, are vital to further investigate cellular kinetics and the relationship with smoking-related changes. This will help to further interpret the current findings in the context of cellular kinetics and could help to improve our understanding of the reversal of smoking-associated disease risk in the future as well as model when and by what mechanism epigenetic alterations return to baseline after smoking cessation.

The impact of e-cigarettes on health and disease risk has not been completely clarified, and conflicting evidence and opinions exist. A 2015 report by Public Health England estimated that electronic cigarettes are at least “95% less harmful” than smoking (6), whereas a 2018 advisory by the U.S. Surgeon General stated the recent surge in e-cigarette use among youth is a “cause for great concern,” in part due to the impact of lifelong nicotine addiction (https://www.cdc.gov/tobacco/e-cigarettes/index.html). Additional studies have since acknowledged potential risks of e-cigarette use such as long-term addiction and a possible link to cancer (64), for example, due to evidence provided by a study by Lee and colleagues, which indicated that e-cigarette smoke damages DNA and reduces repair activity in the mouse heart, lung, and bladder, as well as human lung and bladder cells (8). Moreover, e-cigarette smoke exposure can induce features of chronic obstructive pulmonary disease, a disease associated with smoking, in a nicotine-dependent manner (65), and more recent studies have suggested that e-cigarette smoke can dysregulate immune function and reduce pathogen resistance (66), such as oral cell clearance of potentially pathogenic microbe *Staphylococcus aureus* (67). Our data derived from saliva samples of e-cigarette users suggest epigenetic alterations of directly exposed epithelial cells are, in part, similar to those of cigarette smokers (Fig. 4A and B) and shared sites are enriched for genes involved in DNA damage repair, growth signaling, oxidation, and response to cellular stress, including genes such as *HDAC7*, *MTOR*, *NFE2L2* and *GPX2/3* (Supplementary Fig. S11). Mean methylation at sites involved in detoxification exhibited a duration-dependent effect (Fig. 4D), and was only significantly different from controls following ≥ 1 year of e-cigarette use. Our findings stand in contrast with those of a previous study that observed distinct DNAme patterns of cigarette and e-cigarette users (23). This discrepancy is most likely explained by the different approach applied in this study, especially the identification of cell type–specific DNAme changes.

Smokeless tobacco is another alternative to smoking previously linked to the development of head and neck cancers and other adverse health outcomes. Our data indicate smokeless tobacco use induces similar effects on CpGs in the epithelial hypoM and proximal epithelial hyperM sets as cigarette smoking, but we did not observe any significant effects on immune cells (Fig. 4E and F).

Comparing the three modes of smoking and/or tobacco use (cigarettes, e-cigarettes, or smokeless tobacco), our data suggest that tobacco-containing products (cigarette smoking or smokeless tobacco), or e-cigarette use for more than 1 year, may elicit loss of methylation in epithelial hypoM regions that are associated with detoxification (of tobacco; Fig. 2C). Discontinuation of smoking resulted in a complete reversal of epithelial hypoM alterations (Fig. 3A–C), although the exact timeline and mechanism underlying this reversal is unclear. Only cigarette smokers exhibited alterations in mean DNAme at immune hypoM sites whereas all three types of smoking-related products—cigarettes, e-cigarettes, and smokeless tobacco—elicited proximal epithelial hypermethylation (Fig. 4). Importantly, proximal epithelial hypermethylation was the most consistently associated set of CpGs with lung cancer progression and was strongly altered also in cervical cancer compared with normal cervical tissue (Fig. 5), highlighting a potential link of these sites to carcinogenesis.

Efforts to reduce lung cancer mortality via early detection, such as with low-dose CT in smokers, exist but are likely to require prior risk stratification to reduce false positives (68, 69). Previous studies have demonstrated that methylation at certain sites (18) or composite methylation risk scores (70) can identify individuals at risk of lung cancer in blood samples. The immune hypoM signature did not provide a significant benefit compared with individual methylation levels at *AHRR* or *F2RL3* in blood samples (Fig. 6A and B). Use of buccal or saliva samples could improve convenience for participants and/or reduce healthcare provider costs (e.g., by enabling self-sampling at home). Our data indicate that DNAme at proximal epithelial hyperM sites may be able to detect cancers up to 22 years in the future with an AUC of 0.71 (Fig. 6C). However, given the limited sample size and wide CIs, future prospective sample collections should address whether these sites, or a more informative subset thereof, possibly with a higher AUC, may provide a clinical benefit for stratification.

Our study has several strengths. To our knowledge, our study is one of the first to investigate smoking-associated epigenetic alterations in diverse tissues applying cell type–specific methylation inference to identify differences in epithelial and immune cells. By not limiting our investigation to sources of immune cells (blood) and accounting for cell type–specific differences within proximal and distal sites, the interpretability of our findings is improved and we, for the first time, identify cell type–specific differences in DNAme alterations between epithelial and immune cells in response to smoking. A majority of our reported CpGs (60%) have not previously been described in the literature (Supplementary Fig. S6D), and our observations are validated in several independent datasets (Fig. 3 and 4). We also developed an algorithm to correct for cellular heterogeneity in samples to infer methylation in “pure” epithelial or immune populations of the given sample (Supplementary Fig. S8; Supplementary Data S1). Moreover, we compare alternatives to cigarette smoking and identify similar patterns of DNAme-associated alterations (Fig. 4) and investigate the link of these signatures with progression to cancer (Fig. 5) and cancer prediction (Fig. 6).

Likewise, our study also has limitations. As non-directly exposed epithelial cells are more challenging to obtain in men, we have used only samples from women in our discovery set, which may induce a gender bias. However, the fact that our signatures validate across several independent datasets across both sexes, including a dataset consisting entirely of samples from men (“smokeless tobacco use set”), suggests our findings are applicable to both men and women, although future studies should investigate sex-specific effects.

In absence of large-scale single-cell DNAme data, we utilize bulk DNAme deconvolution and linear models to identify cell-specific smoking-related alterations. Several deconvolution approaches exist (71), including reference-based methods that rely on knowledge of main constituent cell types of the tissue with reference molecular profiles, reference-free methods, or Bayesian approaches, for instance leveraging prior knowledge of distributions of cell types in the studied tissue such as BayesCCE (72). The best deconvolution approach depends on the study type and context (71). We justify the use of the reference-based EpiDISH method with the fact that the main cell types were known and the approach has been previously validated for the sample types assessed in this study (73). We then applied linear models to identify differences across groups and cell types. While these models relied on strong assumptions, previous studies indicated that this approach is feasible and can add additional information (33, 43), and importantly, a separate benchmarking study indicated that linear regression is a valid statistical methodology for DNAme despite the fact that the data do not always perfectly satisfy the assumptions (54). We note that a degree of heteroskedasticity is expected in the case of cell type–specific differential methylation (associated with differential variability). Further work using different deconvolution approaches, Bayesian models that can deal with decomposition of DNAme variability at different levels, for example, refs. 74, 75, and importantly, future studies with other molecular technologies such as single-cell DNAme profiling will undoubtedly be pivotal to validate our findings. This will also be important to evaluate DNAme changes in stem cells to evaluate changes in response to cessation (i.e., evaluation of methylation changes or cell replenishment).

Our study only investigated the association with *cis* gene expression based on available matched methylation and expression data. Future studies should more thoroughly investigate the link between methylation levels and *cis* and *trans* gene expression and protein levels in more detail, for instance using single-cell multiomic analysis. Our sample numbers for the cancer prediction in buccal samples are small. We could not identify any larger sets of buccal or saliva samples with longer-term follow-up in population-based studies, as most studies primarily focus on blood samples. For further clarification of dynamic alterations of smoking, longitudinal samples during smoking cessation would have been valuable. While we were not able to include these in the current study, both aspects are part of ongoing work (ClinicalTrials.gov NCT05678426). It would have been interesting to further dissect whether differences exist between cell subtypes within each tissue (different types of epithelial or immune cells), but this was not possible due to a limited number of samples, and would be best addressed using single-cell DNAme profiling, or bulk DNAme profiling on sorted cells.

In conclusion, our data provide a first insight into cell type–specific epigenetic changes in response to cigarette smoking and highlight that certain epigenetic responses are shared by e-cigarette use, smoking, and cancer. These changes may also be predictive of lung cancer. Future studies to investigate longitudinal dynamics, underlying mechanism, and clinical potential of these signatures are warranted.

## Supplementary Material

Supplementary Information Supplementary Information

Table S1 Supplementary Table 1

Table S2 Supplementary Table 2

Table S3 Supplementary Table 3

Table S4 Supplementary Table 4

Table S5 Supplementary Table 5

Table S6 Supplementary Table 6

Table S7 Supplementary Table 7

Table S8 Supplementary Table 8

Table S9 Supplementary Table 9

Table S10 Supplementary Table 10

Table S11 Supplementary Table 11

Figure S1 Supplementary Figure 1

Figure S2 Supplementary Figure 2

Figure S3 Supplementary Figure 3

Figure S4 Supplementary Figure 4

Figure S5 Supplementary Figure 5

Figure S6 Supplementary Figure 6

Figure S7 Supplementary Figure 7

Figure S8 Supplementary Figure 8

Figure S9 Supplementary Figure 9

Figure S10 Supplementary Figure 10

Figure S11 Supplementary Figure 11

Figure S12 Supplementary Figure 12

Figure S13 Supplementary Figure 13

Supplementary Movie 1 Supplementary Movie 1
