# Supplementary material for: Cigarette Smoking and E-cigarette Use Induce Shared DNA Methylation Changes Linked to Carcinogenesis
Source: Cancer Res. 2024 Mar 19;84(11):1898–914. doi: 10.1158/0008-5472.CAN-23-2957 (PMC11148547; doi:10.1158/0008-5472.CAN-23-2957)
Supplement: Supplementary Information [file can-23-2957_supplementary_information_suppsi.pdf]

Supporting information for

Cigarette smoking and e-cigarette use induce shared DNA methylation changes linked to carcinogenesis

Chiara Herzog, Allison Jones, Iona Evans, Janhavi R. Raut, Michal Zikan, David Cibula, Andrew Wong, Hermann Brenner, Rebecca C. Richmond, and Martin Widschwendter

Martin Widschwendter
email: martin.widschwender@uibk.ac.at

This PDF file includes:

Figures S1 to S13
Tables S1 to S11
Information for Supplementary Movie 1

Contents

Supplementary Figures

1 Supplementary Figure 1. Analysis workflow overview.

2 Supplementary Figure 2. Manhattan and qq-plots for smoking-associated CpGs in buccal samples.

3 Supplementary Figure 3. Manhattan and qq-plots for smoking-associated CpGs in blood samples.

4 Supplementary Figure 4. Manhattan and qq-plots for smoking-associated CpGs in cervical samples.

5 Supplementary Figure 5. Clustering results for cell-specific delta-beta values of significant CpGs using two different approaches.

6 Supplementary Figure 6. Polycomb group target enrichment in CpGs in the four sets, association with expression, and overlap with previously identified sites.

7 Supplementary Figure 7. Epigenetically inferred cell type composition in datasets used in this study.

8 Supplementary Figure 8. Correction of mean beta values for cell type heterogeneity.

9 Supplementary Figure 9. Correlation of corrected mean beta values with smoking pack years in the validation set (buccal and blood samples).

10 Supplementary Figure 10. E-cigarette dataset raw and corrected methylation values and correlation of corrected scores with cigarette and e-cigarette use.

11 Supplementary Figure 11. E-cigarette differential methylation at individual loci and gene set enrichment.

12 Supplementary Figure 12. Smokeless tobacco use dataset raw and corrected methylation values.

13 Supplementary Figure 13. Dependence of mean methylation values on immune cell composition in cancer tissue and carcinoma in situ lesions.

Supplementary Tables

14 Supplementary Table 1. Sample overview

15 Supplementary Table 2. Overview of CpGs associated with hyper- or hypomethylation in smokers compared to never smokers in at least one of the tissues or cell types.

16 Supplementary Table 3. Gene ontology and pathway enrichment for epithelial hypoM.

17 Supplementary Table 4. Gene ontology and pathway enrichment for immune hypoM.

18 Supplementary Table 5. Gene ontology and pathway enrichment for distal epithelial hyperM.

19 Supplementary Table 6. Gene ontology and pathway enrichment for proximal epithelial hyperM.

20 Supplementary Table 7. Association of CpG methylation with gene expression in matched TCGA-LUAD and LUSC data.

21 Supplementary Table 8. Gene ontology and pathway enrichment for sites overlapping between cigarette smokers and e-cigarette users in the epithelial hypoM set.

22 Supplementary Table 9. Gene ontology and pathway enrichment for sites overlapping between cigarette smokers and e-cigarette users in the proximal epithelial hyperM set.

23 Supplementary Table 10. Population characteristics of the ESTHER Study samples.

24 Supplementary Table 11. Odds ratios of methylation values in the ESTHER study samples.

Supplementary Movie

25 Supplementary Movie 1. Illustration of immune cell correction algorithm.

Supplementary Figures

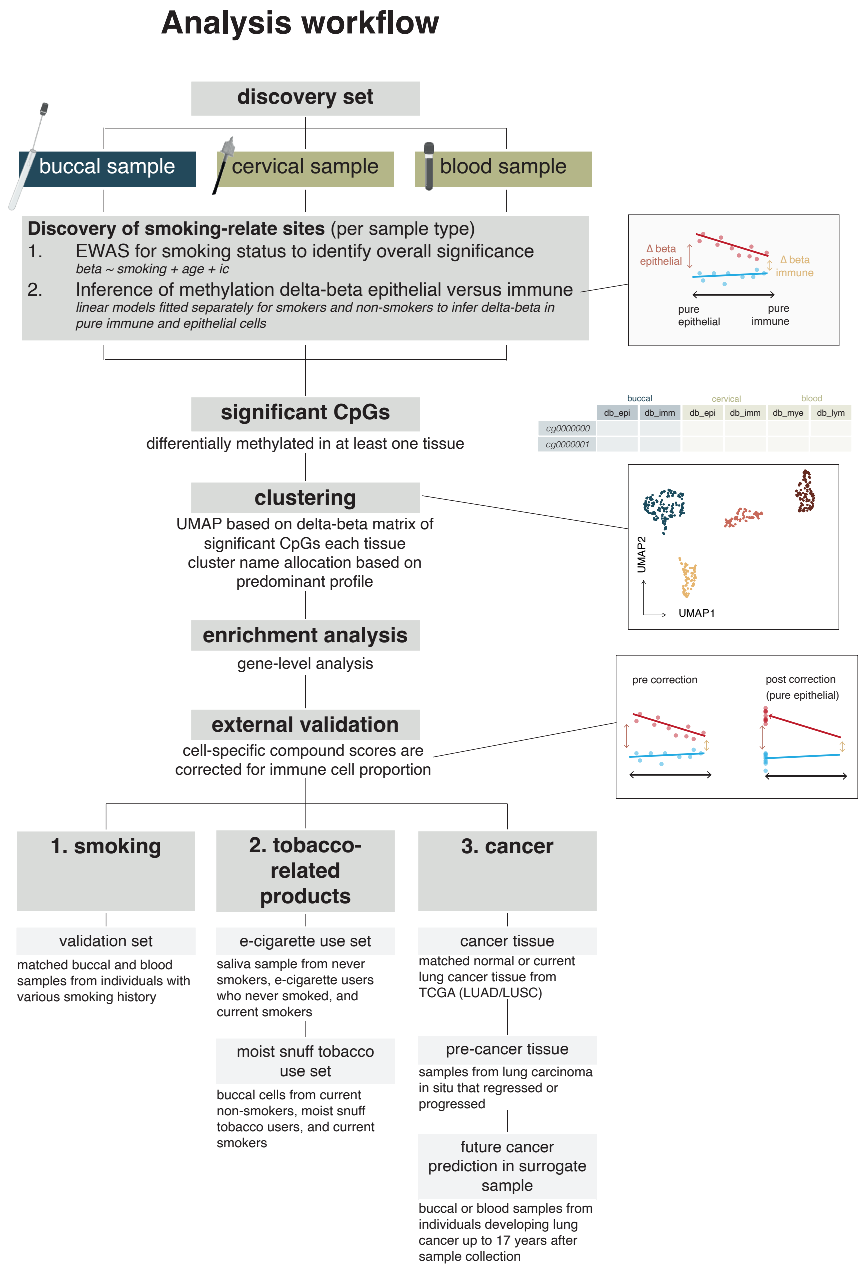

Supplementary Figure 1. Analysis workflow overview.

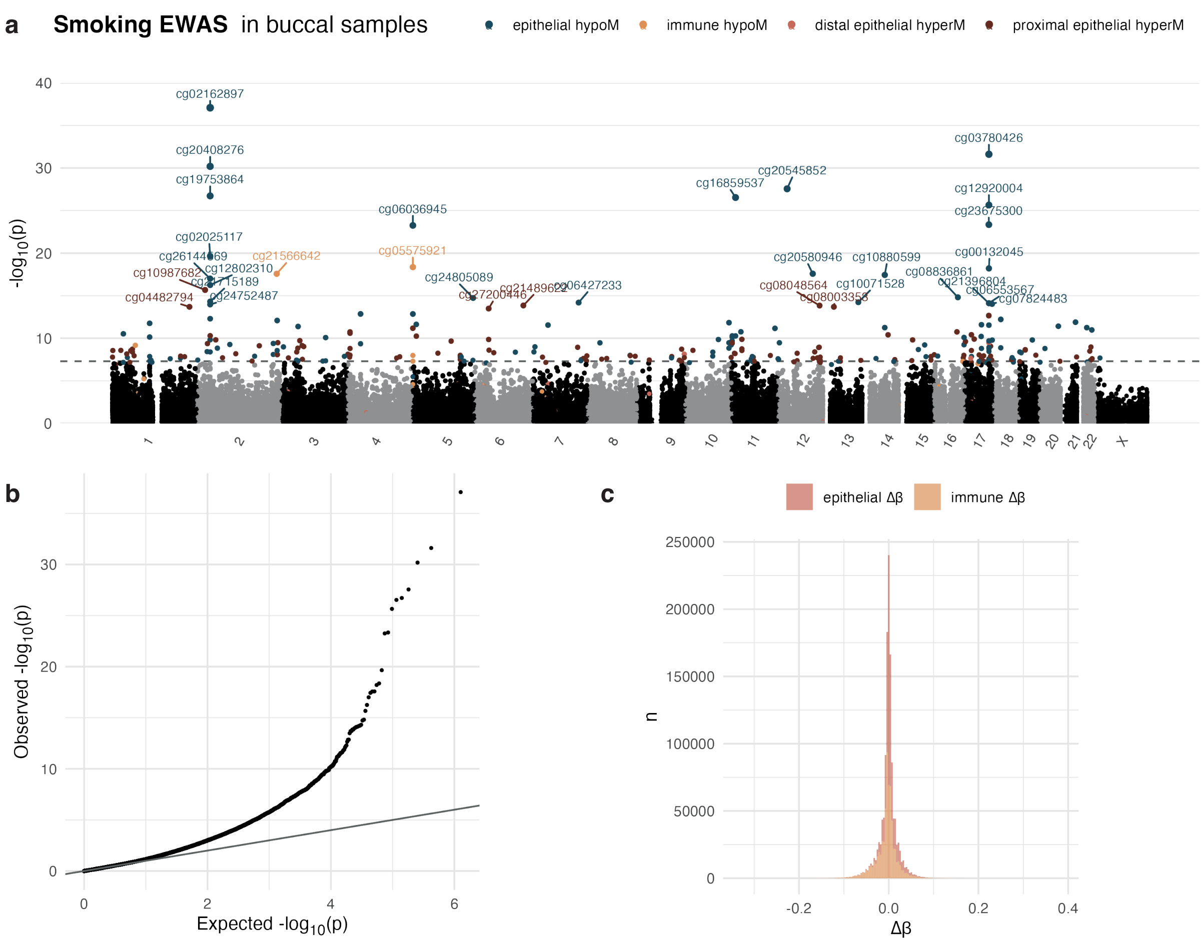

Supplementary Figure 2. Manhattan and qq-plots for smoking-associated CpGs in buccal samples after accounting for age and immune cell proportion. a Manhattan plot for smoking EWAS in buccal samples. CpGs were considered significant if they passed Bonferroni correction (equivalent to p <8.2e-08). **b** qq plot for expected and observed p values in buccal sample EWAS. **c** delta beta (Δ β) values by epithelial and immune fraction in buccal samples.

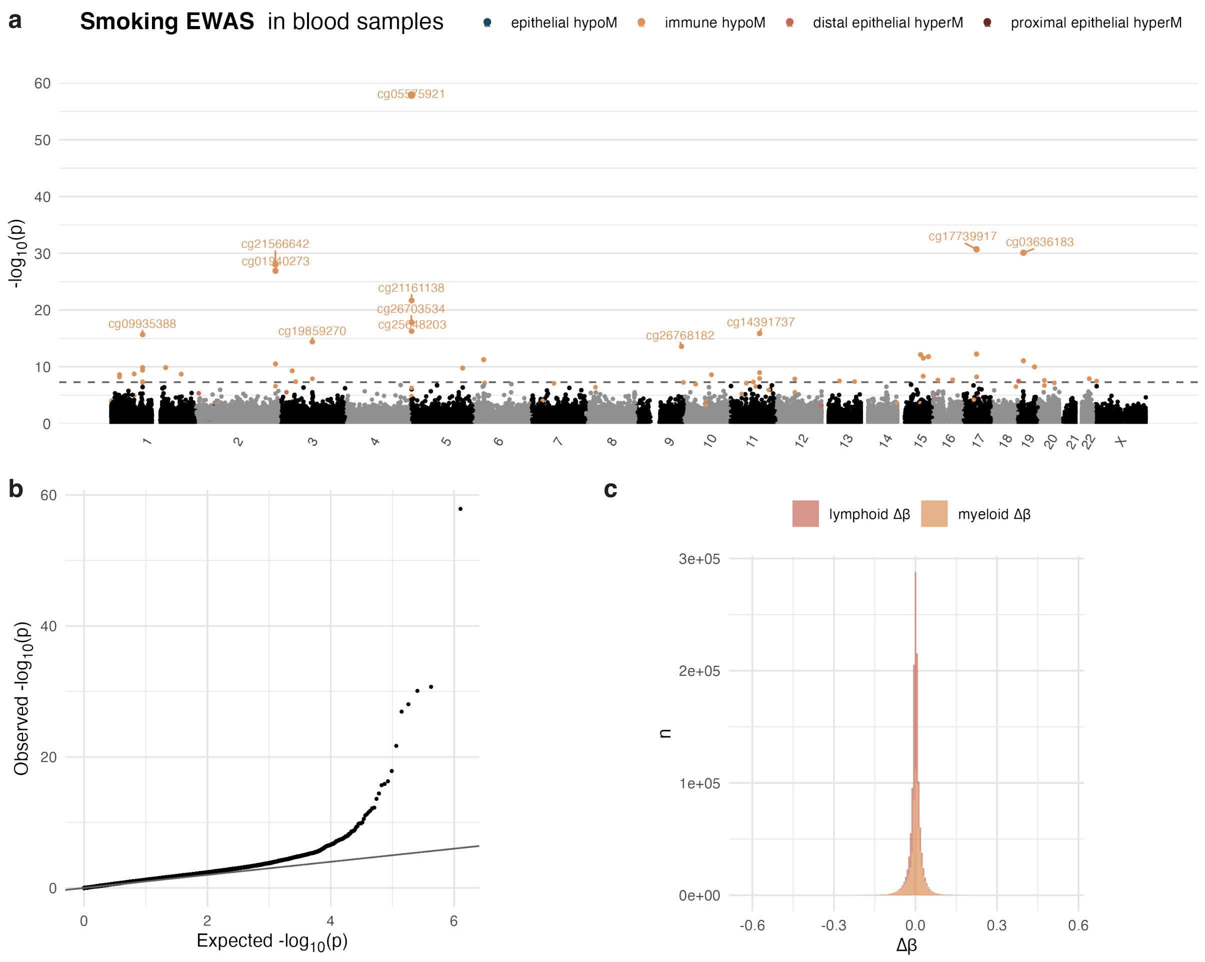

Supplementary Figure 3. Manhattan and qq-plots for smoking-associated CpGs in blood samples after accounting for age and myeloid cell proportion. a Manhattan plot for smoking EWAS in blood samples. CpGs were considered significant if they passed Bonferroni correction (equivalent to $p < 7.9e\text{-}08$). **b** qq-plot for expected and observed p values in blood sample EWAS. **c** delta beta ($\Delta\beta$) values by lymphoid and myeloid fraction in blood samples.

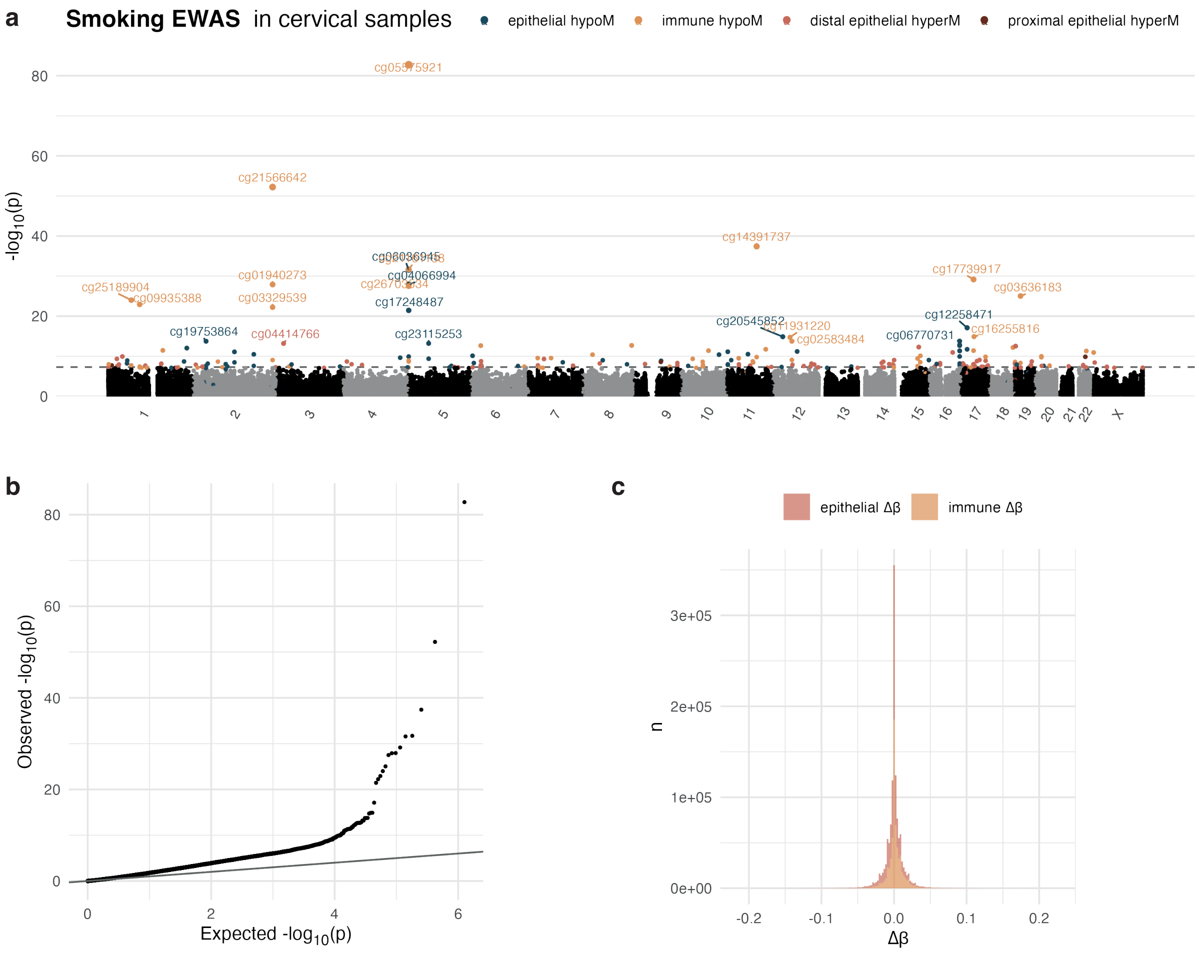

Supplementary Figure 4. Manhattan and qq-plots for smoking-associated CpGs in cervical samples after accounting for age and immune cell proportion. **a** Manhattan plot for smoking EWAS in cervical samples. CpGs were considered significant if they passed Bonferroni correction (equivalent to p <7.9e-08). **b** q-q plot for expected and observed p values in cervical sample EWAS. **c** delta beta (Δ β) values by epithelial and immune fraction in cervical samples.

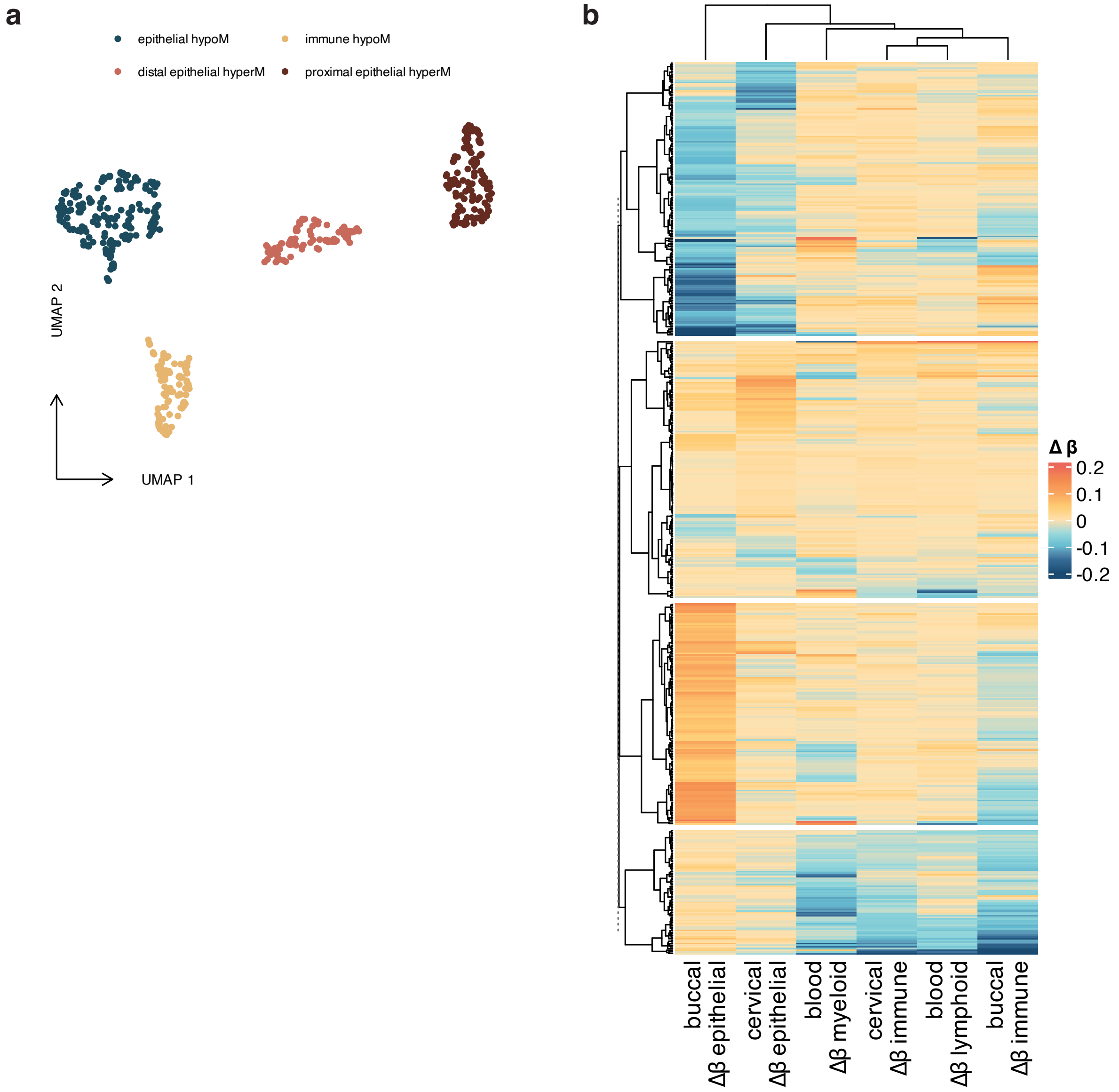

Supplementary Figure 5. Clustering results for cell-specific delta-beta values of significant CpGs using two different approaches. **a** Uniform manifold approximation and projection (UMAP) of the delta-beta of CpGs significantly associated with smoking in at least one tissue indicates the existence of four clusters. Clusters identified here are visualised in Figure 1c and named based on differential methylation in specific tissues and cell types. **b** A second, independent distance-based clustering, utilizing Manhattan distance and Ward's D based on the delta-beta matrix of CpGs significantly associated with smoking in at least one tissue reveals similar clustering as shown in textbf{a} (and Figure 1c).

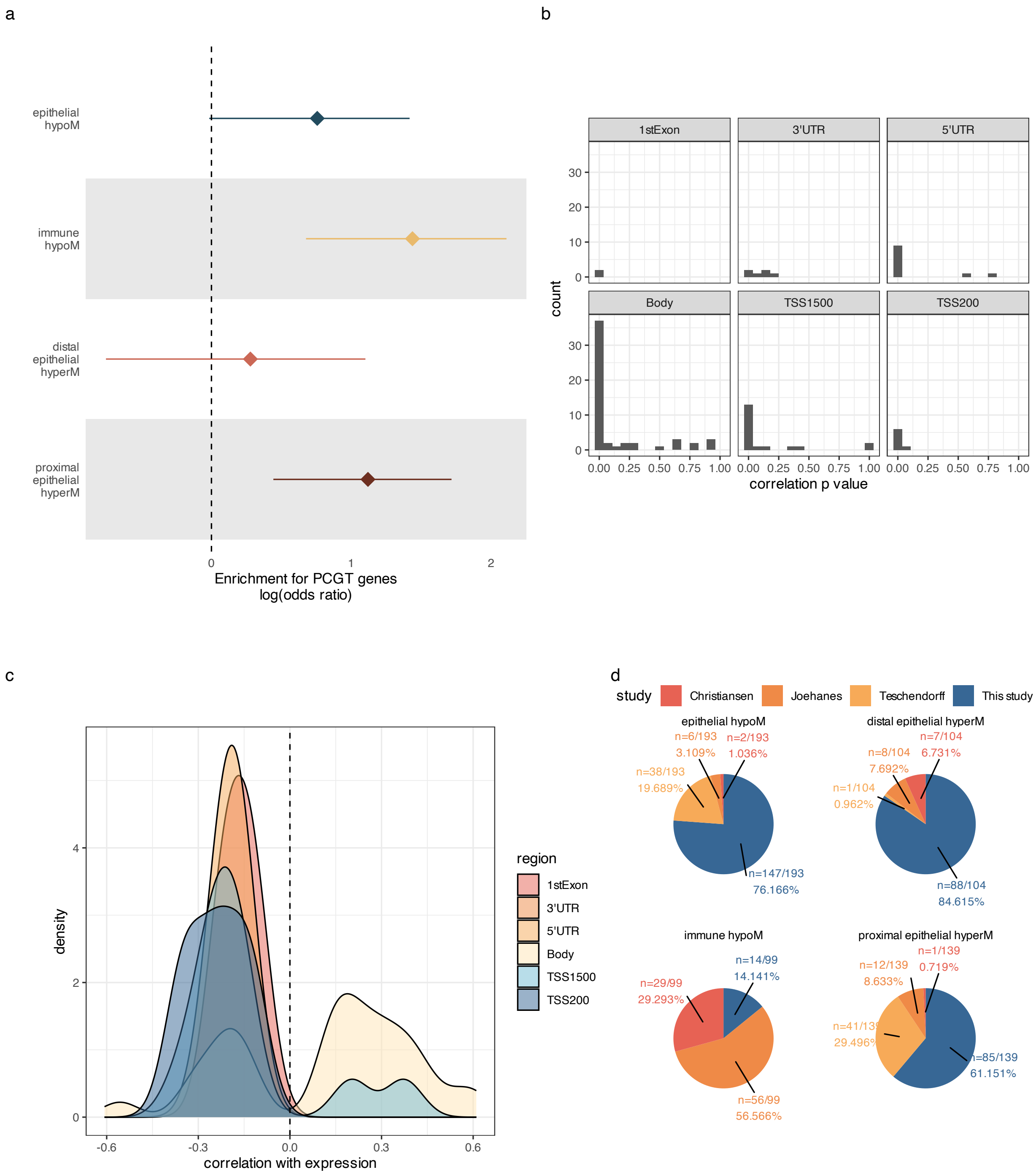

Supplementary Figure 6. Polycomb group target enrichment in CpGs in the four sets, association with expression, and overlap with previously identified sites. a Enrichment for Polycomb group target genes amongst CpGs in the different sets. **b** Histogram of p values for correlation of CpG loci with matched gene expression in TCGA-LUAD and TCGA-LUSC samples, based on genomic location of the CpG. **c** Density plot showing the association of CpG methylation with gene expression by genomic region. Only CpGs that were significantly correlated with expression after Bonferroni correction were included ($p < 0.05$, 55/98). **d** Overlap of CpGs with previously identified smoking CpGs in studies by Joehanes et al., Teschendorff et

al., or Christiansen et al.

a

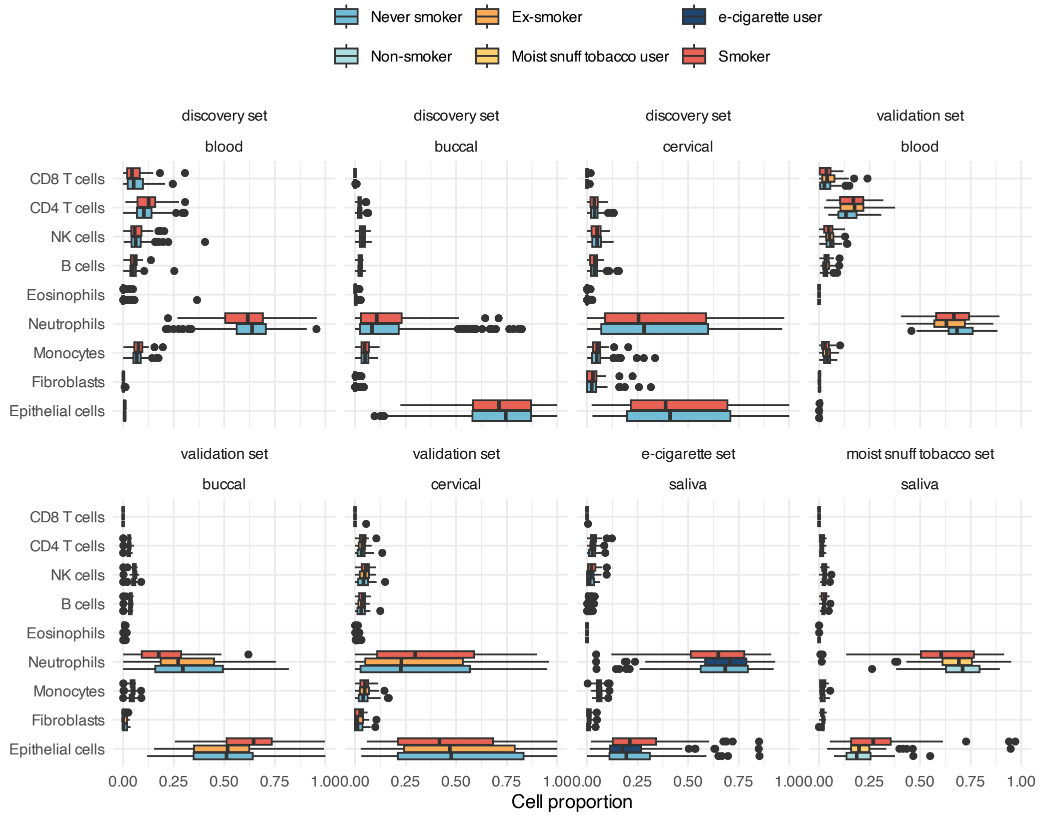

b

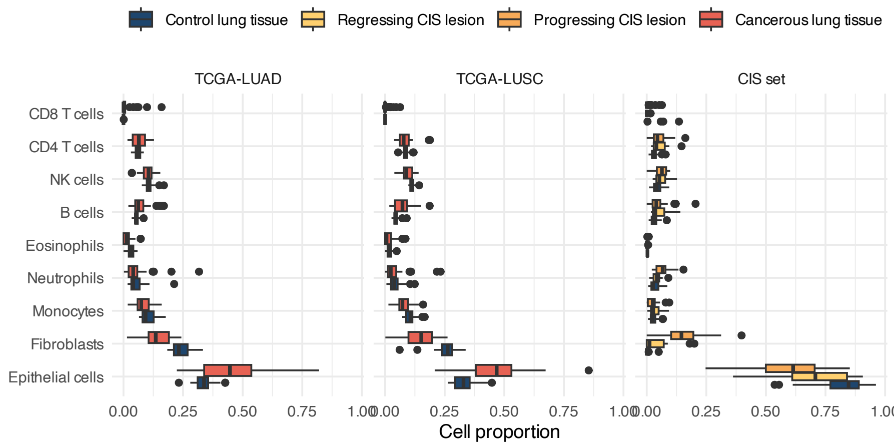

Supplementary Figure 7. Epigenetically inferred cell type composition in datasets used in this study. **a** Inferred cell type proportions in surrogate samples (buccal, blood, cervical) in various datasets included in this study. **b** Inferred cell type proportions in lung tissue datasets. Values were inferred using the EpiDISH package and reference matrices centEpiFibIC.m, and additionally, hierarchical EpiDISH (hEpiDISH) was applied using the centBloodSub.m reference matrix.

Abbreviations: TCGA, The Cancer Genome Atlas. LUAD, lung adenocarcinoma. LUSC, lung squamous cell carcinoma. CIS, carcinoma in situ.

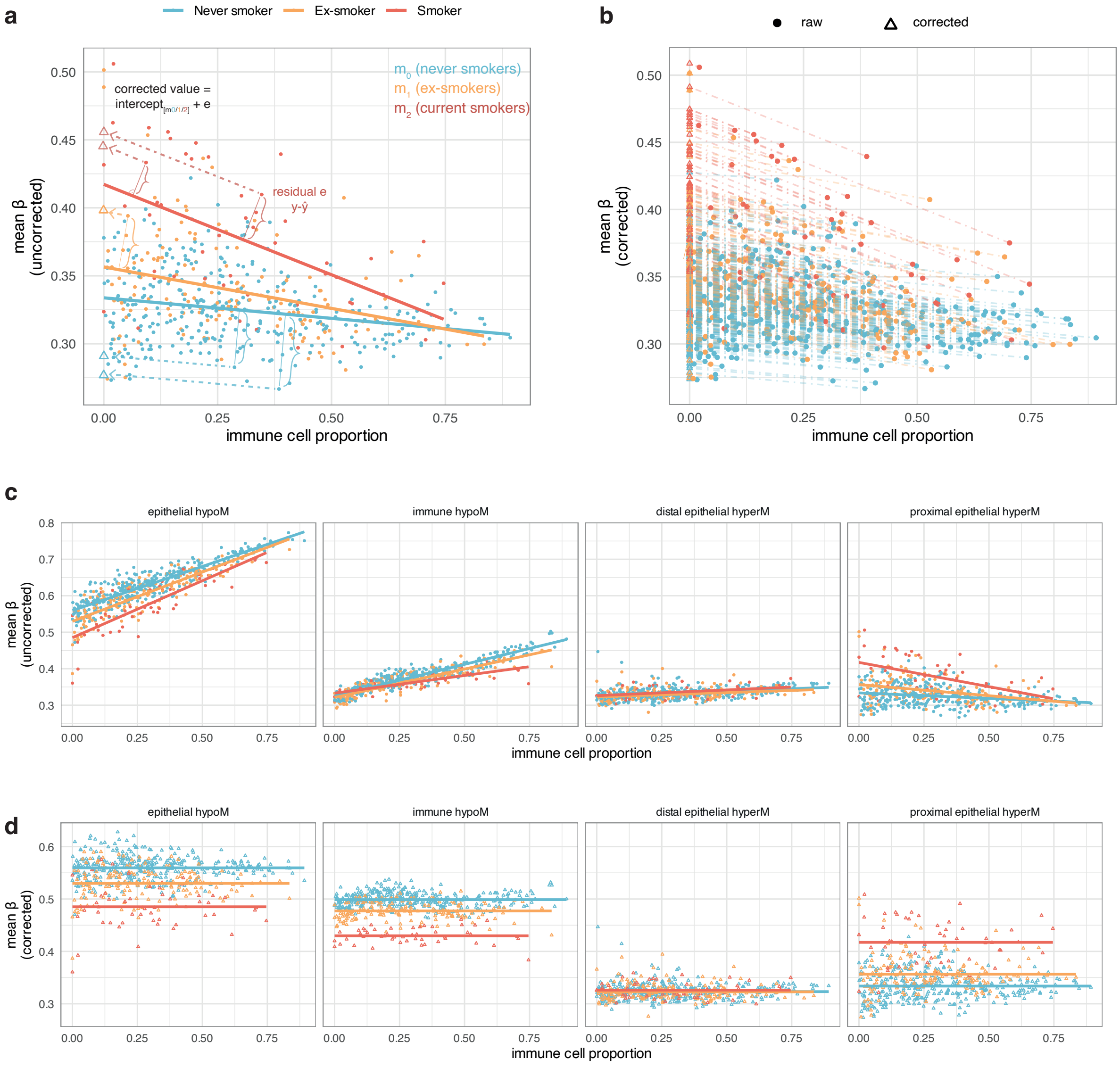

Supplementary Figure 8. Correction of mean beta values for cell type heterogeneity. a Raw mean beta values of proximal epithelial hyperM CpGs (450k) in the discovery set buccal samples. The correction approach is based on slopes and residuals for never, ex- and current smokers by fitting individual linear models for each type. **b** Visualisation of corrected mean beta of values in a, projecting values to immune cell proportion = 0. **c** Raw mean methylation values of all four groups of CpGs in buccal samples in the discovery set. **d** Corrected mean methylation values of all four groups of CpGs in buccal samples in the discovery set.

a

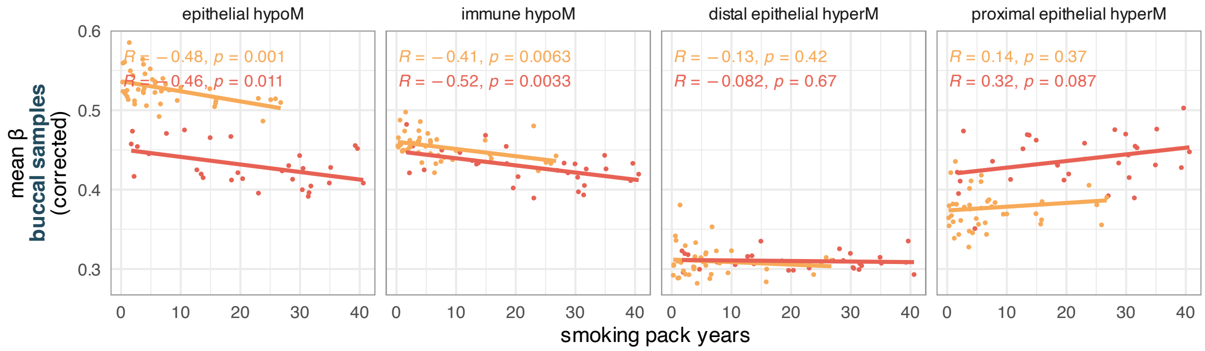

b

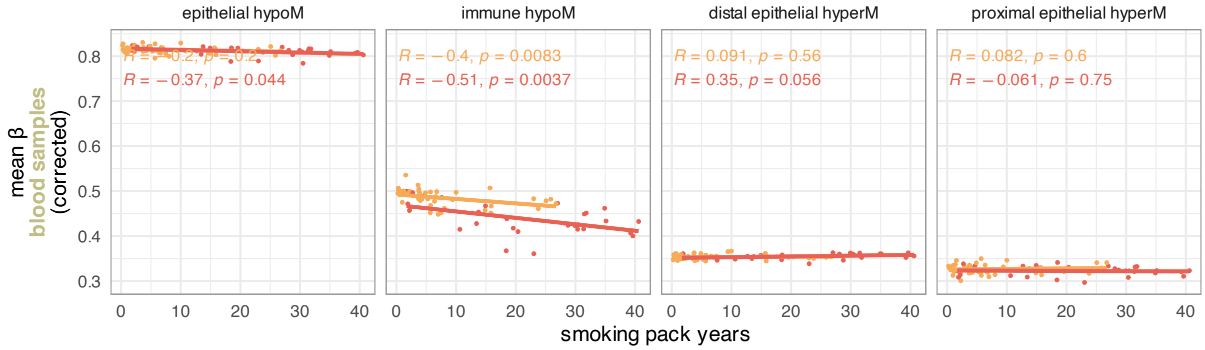

Supplementary Figure 9. Correlation of corrected mean beta values with smoking pack years (buccal and blood samples). For buccal and blood samples, smoking pack year information was available. **a** Pearson correlation of corrected mean beta values of the four groups of CpGs with smoking pack years in buccal samples. **b** Pearson correlation of corrected mean beta values of the four groups of CpGs with smoking pack years in buccal samples.

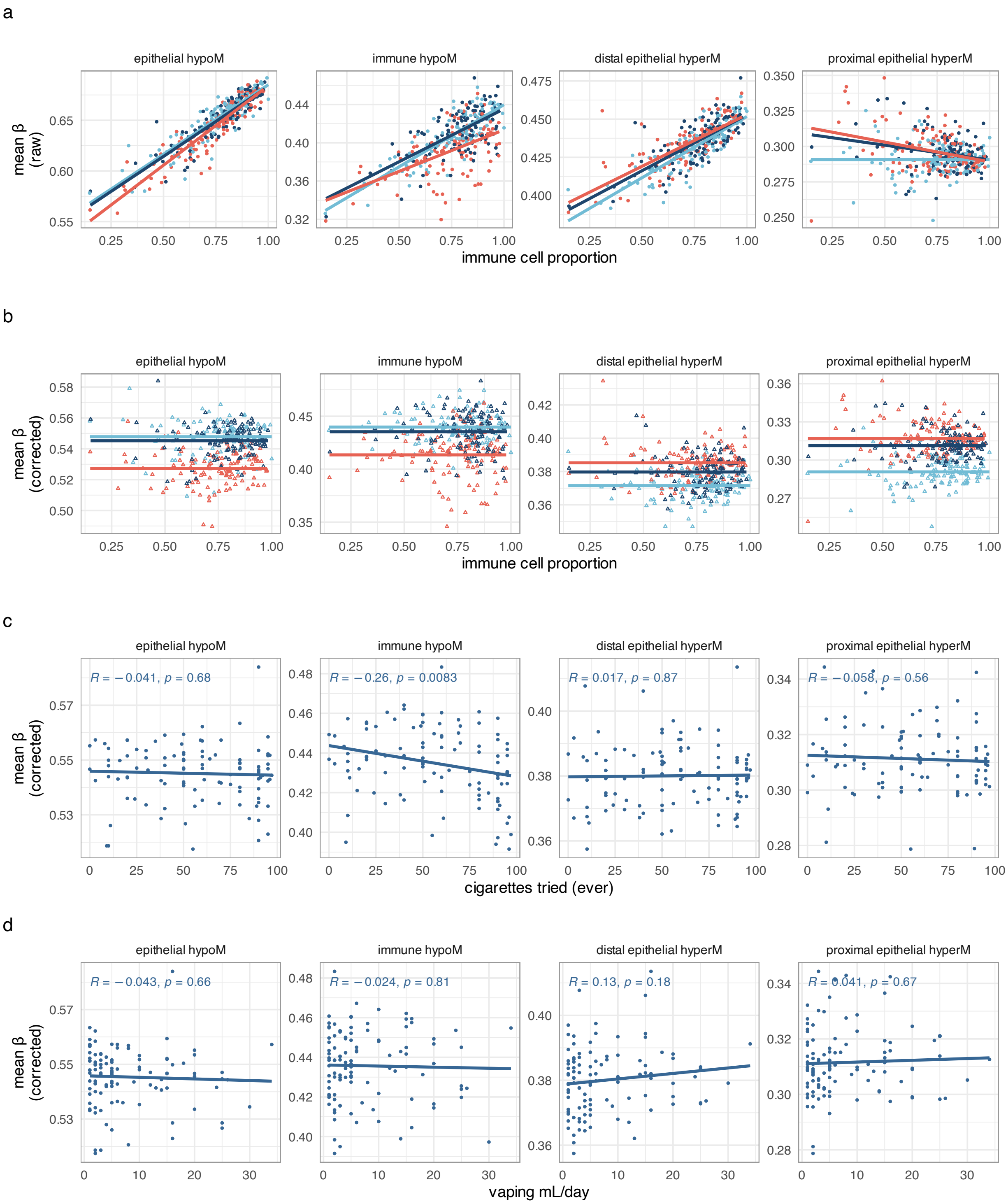

Supplementary Figure 10. E-cigarette dataset raw and corrected methylation values and correlation of corrected scores with cigarette and e-cigarette use. a Raw methylation mean beta values for each group of CpGs. **b** Corrected methylation mean beta values in the same samples. **c** Correlation of methylation beta values and reported number of cigarettes tried (ever) by e-cigarette users. **d** Correlation of methylation beta values and reported mL/day use of e-cigarette liquid.

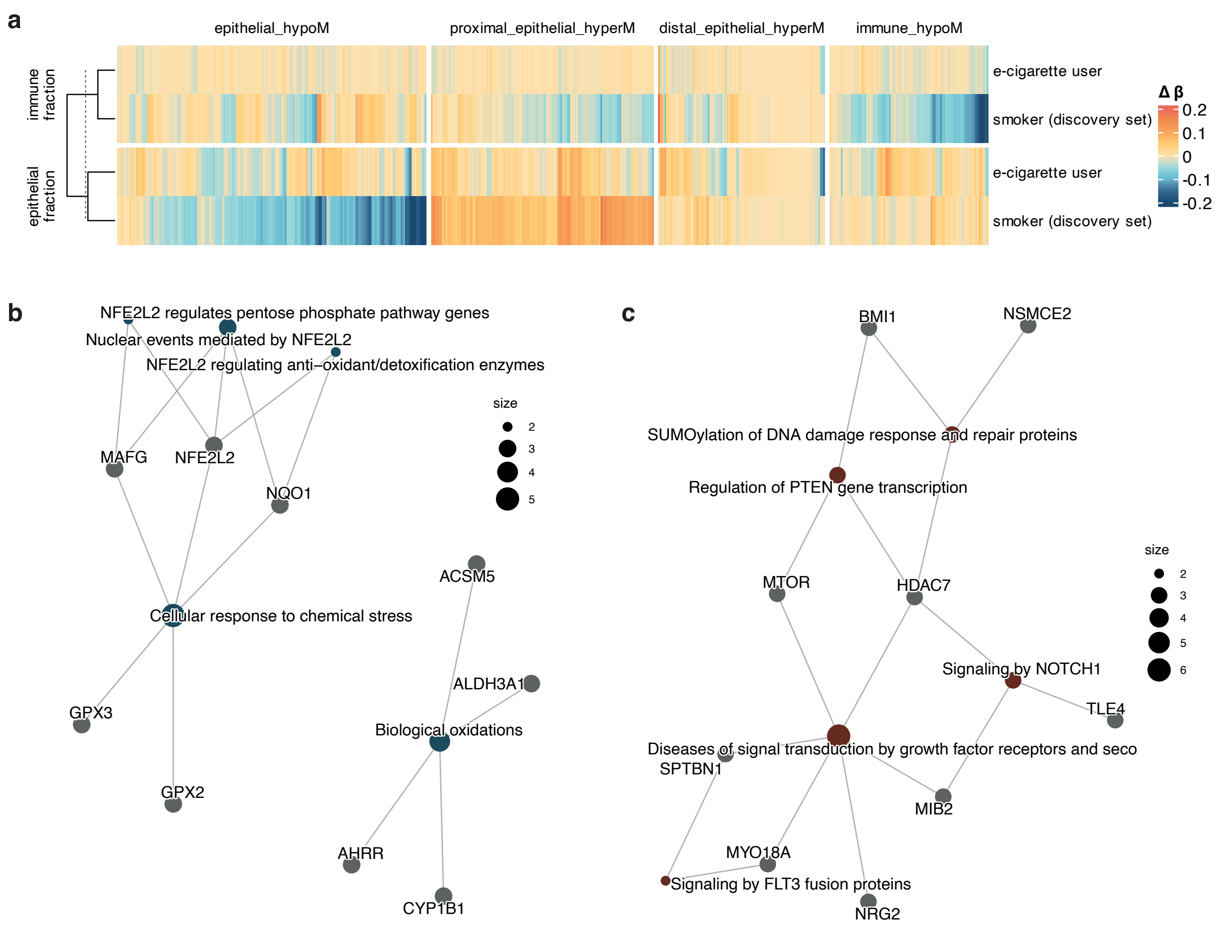

Supplementary Figure 11. E-cigarette differential methylation at individual loci and gene set enrichment. **a** Heatmap comparing delta beta values in the inferred immune and epithelial fractions in buccal samples of the discovery set and e-cigarette users. **b** Reactome pathway enrichment for sites sharing the same directionality in the epithelial fraction of epithelial hypoM as in smokers in the discovery set. **c** Reactome pathway enrichment for sites sharing the same directionality in the epithelial fraction of proximal epithelial hyperM as in smokers in the discovery set.

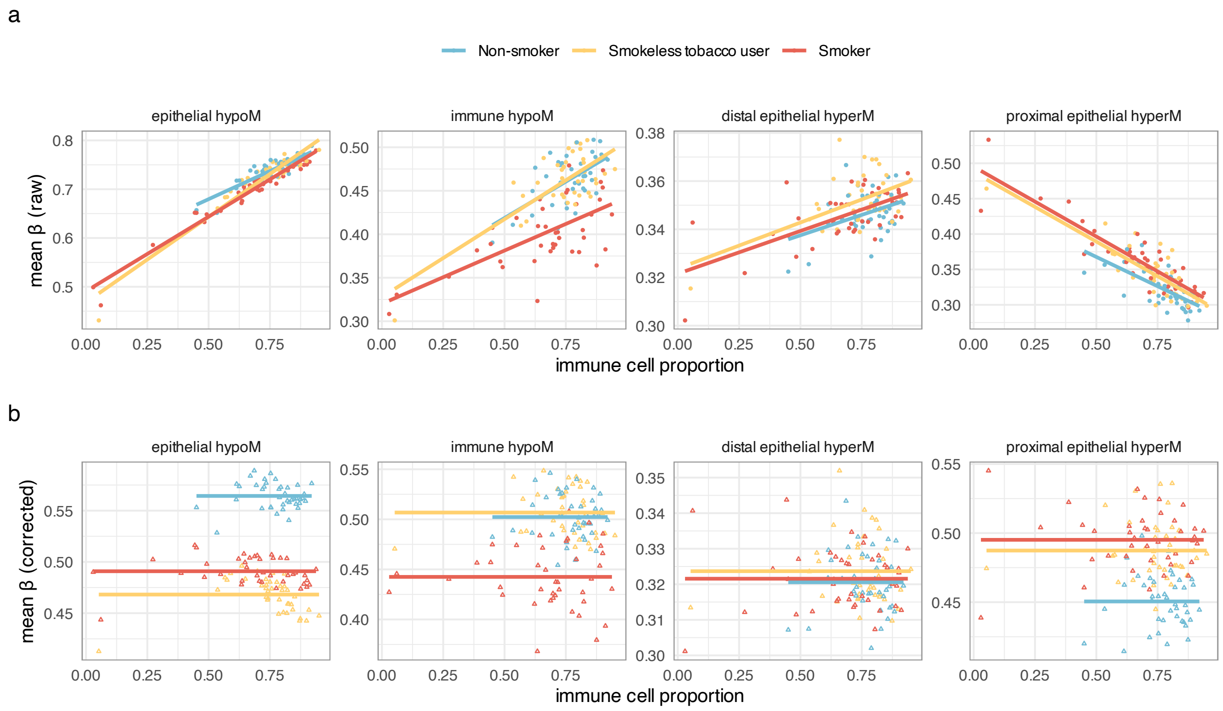

Supplementary Figure 12. Smokeless tobacco use dataset raw and corrected methylation values. a Raw methylation mean beta values for each set of CpGs. **b** Corrected methylation mean beta values in the same samples.

a

b

c

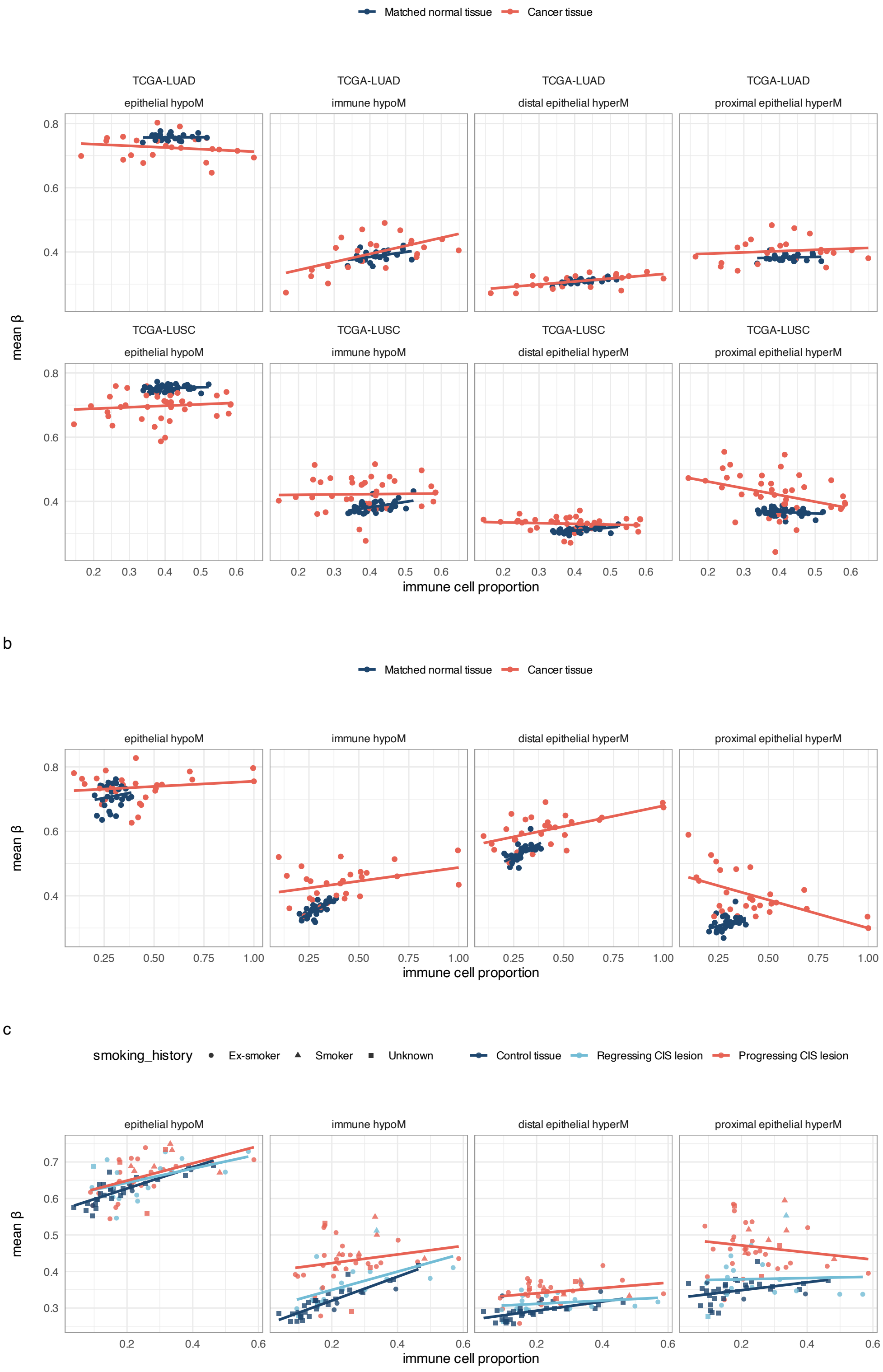

Supplementary Figure 13. Dependence of mean methylation values on immune cell composition in cancer tissue and carcinoma in situ lesions. a Raw methylation mean beta values for each set of CpGs versus inferred immune cell proportion in TCGA-LUAD and TCGA-LUSC samples with matched control tissue. **b** Raw methylation mean beta values for each set of CpGs versus inferred immune cell proportion

in cervical cancer versus normal control tissue. **c** Raw methylation mean beta values for each set of CpGs in control samples or regressing or progressing CIS lesions.

Supplementary Tables

Supplementary Table 1. Sample overview of datasets used to investigate the impact of tobacco, e-cigarette use, or smokeless tobacco, on different cell types.

	discovery set<br>n=2,341	validation set<br>n=304	e-cigarette use set<br>n=350	smokeless tobacco use set<br>n=120
**blood sample**				
Accession	EGAS00001005055	N/A*	-	-
n	464	152	-	-
Platform	EPIC	450K	-	-
Female (%)	464 (100%)	152 (100%)	-	-
Age	46.8 (37-57)	55	-	-
Never smoker	275 (59.3%)	72 (47.4%)	-	-
Ex-smoker	134 (28.9%)	49 (32.2%)	-	-
Smoker	55 (11.9%)	31 (20.4%)	-	-
**buccal sample**				
Accession	EGAS00001005055	N/A*	-	-
n	542	152	-	-
Platform	EPIC	450K	-	-
Female (%)	542 (100%)	152 (100%)	-	-
Age	48.06 (39-57)	55	-	-
Never smoker	333 (61.4%)	72 (47.4%)	-	-
Ex-smoker	159 (29.3%)	49 (32.2%)	-	-
Smoker	50 (9.2%)	31 (20.4%)	-	-
**cervical sample**				
Accession	EGAS00001005055	EGAS00001005055	-	-
n	1335	442	-	-
Platform	EPIC	EPIC	-	-
Female (%)	1335 (100%)	442 (100%)	-	-
Age	47.27 (38-57)	55	-	-
Never smoker	765 (57.3%)	251 (56.8%)	-	-
Ex-smoker	419 (31.4%)	139 (31.4%)	-	-
Smoker	151 (11.3%)	52 (11.8%)	-	-
**saliva sample**				
Accession	-	-	N/A*	GSE94876
n	-	-	350	120
Platform	-	-	EPIC	450K
Female (%)	-	-	143 (40.9%)	0 (0%)
Age	-	-	20 (18-23)	46.5 (40-53)
Never smoker	-	-	117 (33.4%)	-
Non-smoker	-	-	-	40 (33.3%)
Smokeless Tobacco User	-	-	-	40 (0%)
E-cigarette user	-	-	116 (33.1%)	-
Smoker	-	-	117 (33.4%)	40 (33.3%)

* data not publicly deposited due to restrictions on informed consent.

Supplementary Table 2. Overview of CpGs associated with hyper or hypomethylation in smokers compared to never smokers in at least one of the tissues or cell types.

See separate .xlsx file.

Supplementary Table 3. Gene ontology and pathway enrichment for epithelial hypoM.

See separate .xlsx file.

Supplementary Table 4. Gene ontology and pathway enrichment for immune hypoM.

See separate .xlsx file.

Supplementary Table 5. Gene ontology and pathway enrichment for distal epithelial hyperM.

See separate .xlsx file.

Supplementary Table 6. Gene ontology and pathway enrichment for proximal epithelial hyperM.

See separate .xlsx file.

Supplementary Table 7. Association of CpG methylation with gene expression in matched TCGA-LUAD and LUSC data.

See separate .xlsx file.

Supplementary Table 8. Gene ontology and pathway enrichment for sites overlapping between cigarette smokers and e-cigarette users in the epithelial hypoM set.

See separate .xlsx file.

Supplementary Table 9. Gene ontology and pathway enrichment for sites overlapping between cigarette smokers and e-cigarette users in the proximal epithelial hyperM set

See separate .xlsx file.

Supplementary Table 10. Population characteristics of the ESTHER Study samples.

	Characteristics	Controls n=1403	LC cases n=90
**Age at sampling**			
	Mean (SD)	63.5 (6.6)	62.0 (6.0)
	Median (range)	64 (50-75)	62 (50-74)
**Gender– counts (%)**			
	Male	607 (43.3)	60 (66.7)
	Female	796 (56.7)	30 (33.3)
**Time between sampling & diagnosis**			
	Range	--	0.1- 16.8
	Median (interquartile range)	--	9.7 (5.0- 12.5)
**Smoking status at sampling [n (%)]**[1]			
	Never smoker	692 (49.3)	13 (14.4)
	Former smoker	426 (30.4)	26 (28.9)
	Current smoker	242 (17.2)	50 (55.6)
**Smoking pack-years**[2]			
	Mean ± SD	12.1 ± 18.3	32.4 ± 21.2
**Immune_hypoM score**			
	Mean ± SD[3]	0.40 ± 0.02	0.37 ± 0.03

[1] Data missing for 1 lung cancer case and 43 controls (2.9 % of total participants)

[2] Data missing for 6 lung cancer cases and 135 controls (9.4 % of total participants)

[3] $p < 2.2e-16$, as assessed by Mann-Whitney test.

Abbreviations: LC, lung cancer; n, number; SD, standard deviation.

Supplementary Table 11. Odds ratios of methylation values in the ESTHER study samples.

LC cases (n)	Controls (n)	Model 1[1]		Model 2[2]		Model 3[3]	
		OR (95% CI)[4]	p value[4]	OR (95% CI)[4]	p value[4]	OR (95% CI)[4]	p value[4]
84	1268	0.42 (0.35 - 0.51)	**<2e-16**	0.45 (0.37 - 0.55)	**1.32e-15**	0.96 (0.94 - 0.97)	**1.64e-07**

[1] Model 1: without adjustment for any confounders.

[2] Model 2: model 1 plus adjustment for age and sex.

[3] Model 3: model 2 plus adjustment for smoking status and pack-years.

[4] OR per standard deviation increase in the Immune_hypoM score; OR, 95% CI and two-sided p-values were generated from logistic regression model.

Abbreviations: CI, confidence interval; LC, lung cancer; n, number; OR, odds ratio.

Supplementary Movie

Supplementary Movie 1. Illustration of immune cell correction algorithm. Data is derived from Supplementary Figure 8a, b.
