# Supplementary material for: Cigarette Smoking and E-cigarette Use Induce Shared DNA Methylation Changes Linked to Carcinogenesis
Source: Cancer Res. 2024 Mar 19;84(11):1898–914. doi: 10.1158/0008-5472.CAN-23-2957 (PMC11148547; doi:10.1158/0008-5472.CAN-23-2957)
Supplement: Table S1 — Supplementary Table 1 [file can-23-2957_table_s1_suppst1.pdf]

Supplementary Table 1. Sample overview of datasets used to investigate the impact of tobacco, e-cigarette use, or smokeless tobacco, on different cell types.

	discovery set<br><br>n=2,341	validation set<br><br>n=304	e-cigarette use set<br>n=350	smokeless tobacco use set<br>n=120
**blood sample**				
Accession	EGAS00001005055	N/A*	-	-
n	464	152	-	-
Platform	EPIC	450K	-	-
Female (%)	464 (100%)	152 (100%)	-	-
Age	46.8 (37-57)	55	-	-
Never smoker	275 (59.3%)	72 (47.4%)	-	-
Ex-smoker	134 (28.9%)	49 (32.2%)	-	-
Smoker	55 (11.9%)	31 (20.4%)	-	-
**buccal sample**				
Accession	EGAS00001005055	N/A*	-	-
n	542	152	-	-
Platform	EPIC	450K	-	-
Female (%)	542 (100%)	152 (100%)	-	-
Age	48.06 (39-57)	55	-	-
Never smoker	333 (61.4%)	72 (47.4%)	-	-
Ex-smoker	159 (29.3%)	49 (32.2%)	-	-
Smoker	50 (9.2%)	31 (20.4%)	-	-
**cervical sample**				
Accession	EGAS00001005055	EGAS00001005055	-	-
n	1335	442	-	-
Platform	EPIC	EPIC	-	-
Female (%)	1335 (100%)	442 (100%)	-	-
Age	47.27 (38-57)	55	-	-
Never smoker	765 (57.3%)	251 (56.8%)	-	-
Ex-smoker	419 (31.4%)	139 (31.4%)	-	-
Smoker	151 (11.3%)	52 (11.8%)	-	-
**saliva sample**				
Accession	-	-	N/A*	GSE94876
n	-	-	350	120
Platform	-	-	EPIC	450K
Female (%)	-	-	143 (40.9%)	0 (0%)
Age	-	-	20 (18-23)	46.5 (40-53)
Never smoker	-	-	117 (33.4%)	-
Non-smoker	-	-	-	40 (33.3%)
Smokeless Tobacco User	-	-	-	40 (0%)
E-cigarette user	-	-	116 (33.1%)	-
Smoker	-	-	117 (33.4%)	40 (33.3%)

* data not publicly deposited due to restrictions on informed consent.
