# Supplementary material for: Cigarette Smoking and E-cigarette Use Induce Shared DNA Methylation Changes Linked to Carcinogenesis
Source: Cancer Res. 2024 Mar 19;84(11):1898–914. doi: 10.1158/0008-5472.CAN-23-2957 (PMC11148547; doi:10.1158/0008-5472.CAN-23-2957)
Supplement: Table S10 — Supplementary Table 10 [file can-23-2957_table_s10_suppst10.pdf]

Supplementary Table 10. Population characteristics of the ESTHER Study samples.

Characteristics		Controls n=1403	LC cases n=90
**Age at sampling**			
	Mean (SD)	63.5 (6.6)	62.0 (6.0)
	Median (range)	64 (50-75)	62 (50-74)
**Gender– counts (%)**			
	Male	607 (43.3)	60 (66.7)
	Female	796 (56.7)	30 (33.3)
**Time between sampling & diagnosis**			
	Range	--	0.1- 16.8
	Median (interquartile range)	--	9.7 (5.0- 12.5)
**Smoking status at sampling [n (%)]**[1]			
	Never smoker	692 (49.3)	13 (14.4)
	Former smoker	426 (30.4)	26 (28.9)
	Current smoker	242 (17.2)	50 (55.6)
**Smoking pack-years**[2]			
	Mean ± SD	12.1 ± 18.3	32.4 ± 21.2
**Immune_hypoM score**			
	Mean ± SD[3]	0.40 ± 0.02	0.37 ± 0.03

[1] Data missing for 1 lung cancer case and 43 controls (2.9 % of total participants)

[2] Data missing for 6 lung cancer cases and 135 controls (9.4 % of total participants)

[3] $p < 2.2e-16$, as assessed by Mann-Whitney test.

Abbreviations: LC, lung cancer; n, number; SD, standard deviation.
