# Supplementary material for: Cigarette Smoking and E-cigarette Use Induce Shared DNA Methylation Changes Linked to Carcinogenesis
Source: Cancer Res. 2024 Mar 19;84(11):1898–914. doi: 10.1158/0008-5472.CAN-23-2957 (PMC11148547; doi:10.1158/0008-5472.CAN-23-2957)
Supplement: Table S11 — Supplementary Table 11 [file can-23-2957_table_s11_suppst11.pdf]

Supplementary Table 11. Odds ratios of methylation values in the ESTHER study samples.

LC cases (n)	Controls (n)	Model 1[1]		Model 2[2]		Model 3[3]	
		OR (95% CI)[4]	p value[4]	OR (95% CI)[4]	p value[4]	OR (95% CI)[4]	p value[4]
84	1268	0.42 (0.35 - 0.51)	**<2e-16**	0.45 (0.37 - 0.55)	**1.32e-15**	0.96 (0.94 - 0.97)	**1.64e-07**

[1] Model 1: without adjustment for any confounders.

[2] Model 2: model 1 plus adjustment for age and sex.

[3] Model 3: model 2 plus adjustment for smoking status and pack-years.

[4] OR per standard deviation increase in the Immune_hypoM score; OR, 95% CI and two-sided p-values were generated from logistic regression model.

Abbreviations: CI, confidence interval; LC, lung cancer; n, number; OR, odds ratio.
