# Supplementary figures and images for: Cigarette Smoking and E-cigarette Use Induce Shared DNA Methylation Changes Linked to Carcinogenesis
Source: Cancer Res. 2024 Mar 19;84(11):1898–914. doi: 10.1158/0008-5472.CAN-23-2957 (PMC11148547; doi:10.1158/0008-5472.CAN-23-2957)

## Analysis workflow

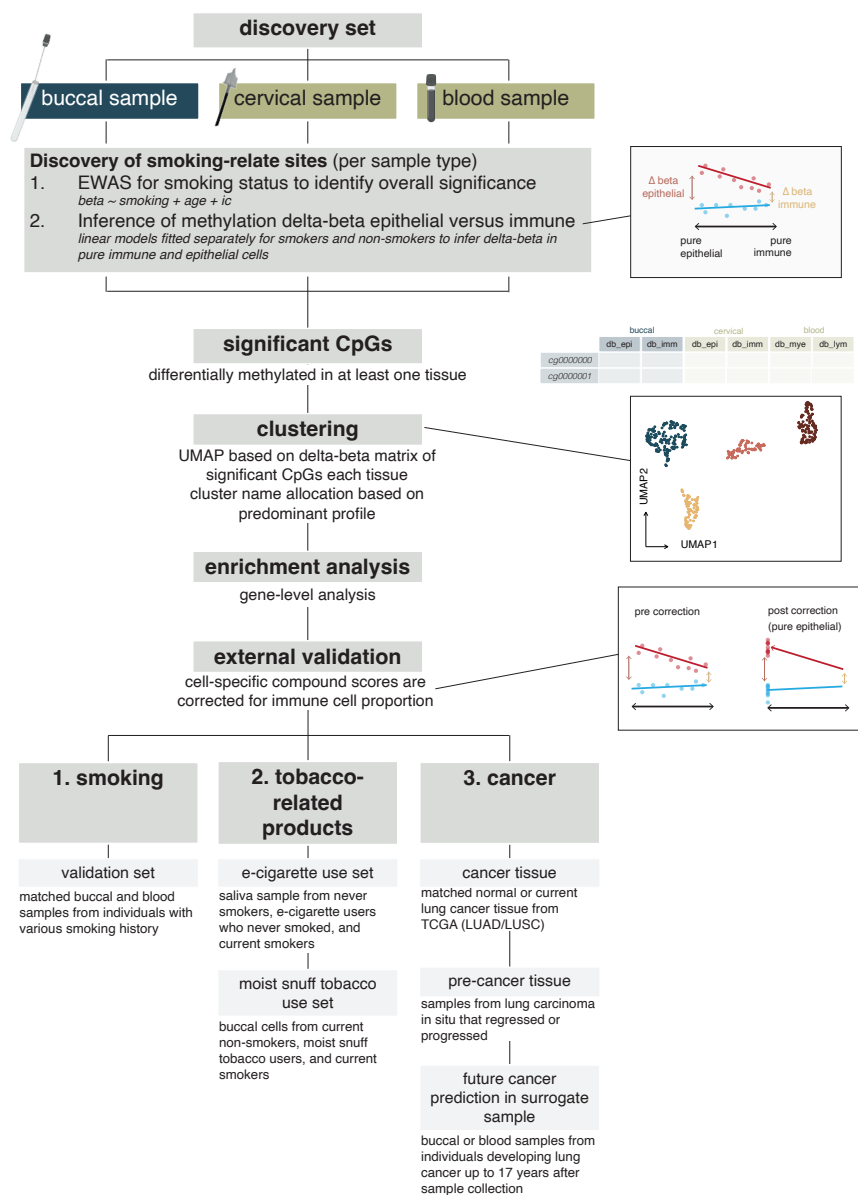

Supplementary Figure 1. Analysis workflow overview.

Supplement: Figure S1 — Supplementary Figure 1 [file can-23-2957_figure_s1_suppsf1.pdf]
