# Supplementary material for: Cigarette Smoking and E-cigarette Use Induce Shared DNA Methylation Changes Linked to Carcinogenesis
Source: Cancer Res. 2024 Mar 19;84(11):1898–914. doi: 10.1158/0008-5472.CAN-23-2957 (PMC11148547; doi:10.1158/0008-5472.CAN-23-2957)
Supplement: Figure S2 — Supplementary Figure 2 [file can-23-2957_figure_s2_suppsf2.pdf]

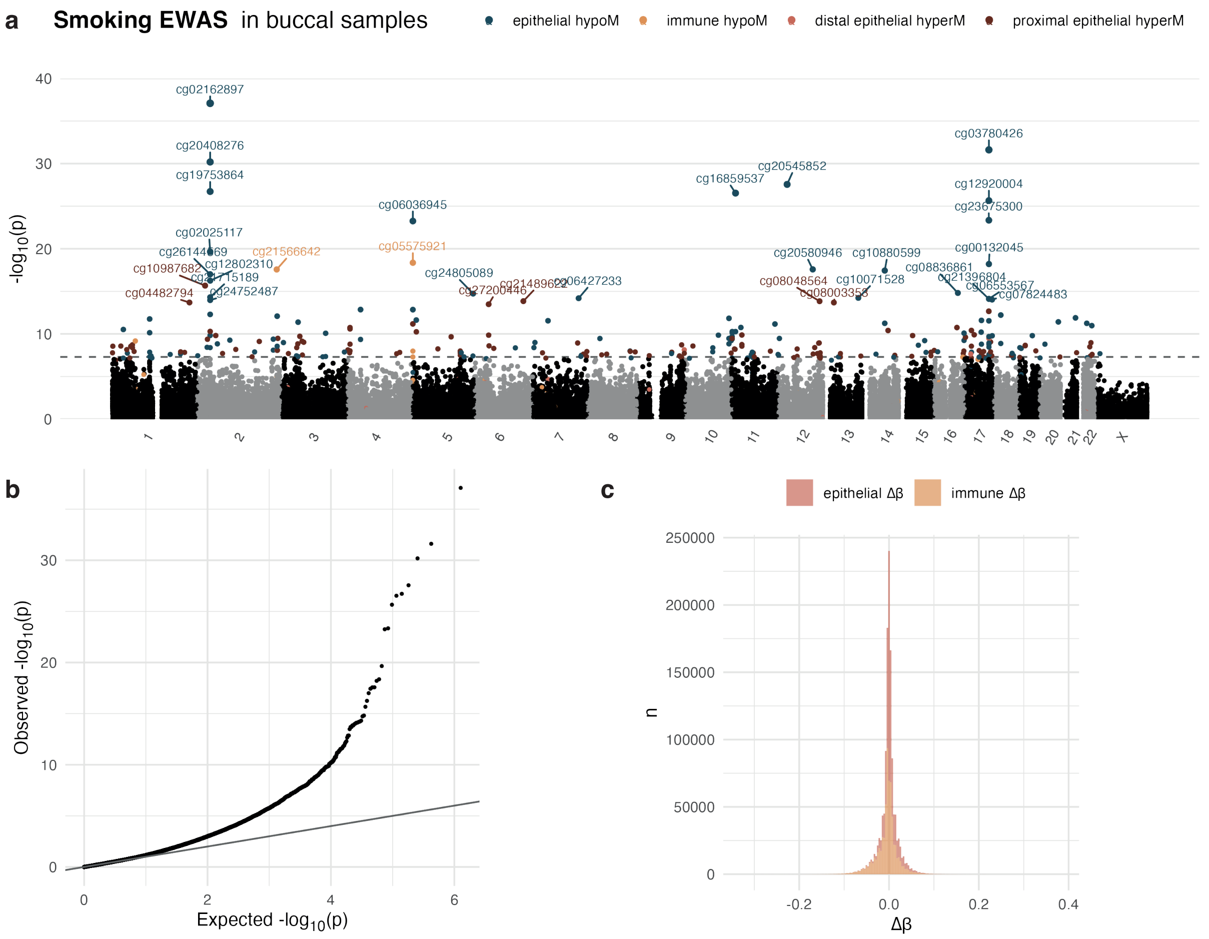

Supplementary Figure 2. Manhattan and qq-plots for smoking-associated CpGs in buccal samples after accounting for age and immune cell proportion. a Manhattan plot for smoking EWAS in buccal samples. CpGs were considered significant if they passed Bonferroni correction (equivalent to p <8.2e-08). **b** qq plot for expected and observed p values in buccal sample EWAS. **c** delta beta ($\Delta\beta$) values by epithelial and immune fraction in buccal samples.
