# Supplementary material for: Cigarette Smoking and E-cigarette Use Induce Shared DNA Methylation Changes Linked to Carcinogenesis
Source: Cancer Res. 2024 Mar 19;84(11):1898–914. doi: 10.1158/0008-5472.CAN-23-2957 (PMC11148547; doi:10.1158/0008-5472.CAN-23-2957)
Supplement: Figure S3 — Supplementary Figure 3 [file can-23-2957_figure_s3_suppsf3.pdf]

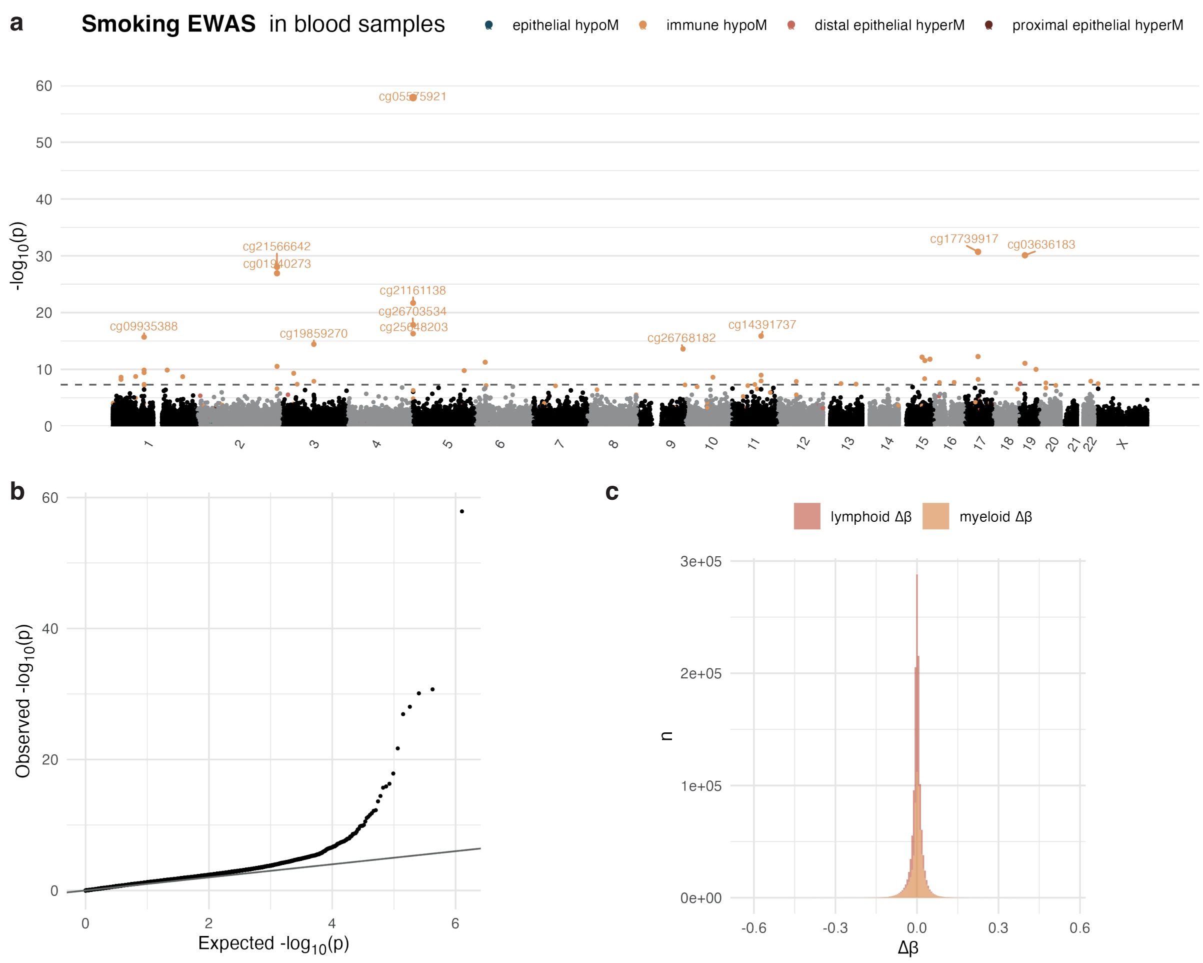

Supplementary Figure 3. Manhattan and qq-plots for smoking-associated CpGs in blood samples after accounting for age and myeloid cell proportion. a Manhattan plot for smoking EWAS in blood samples. CpGs were considered significant if they passed Bonferroni correction (equivalent to p <7.9e-08). **b** qq-plot for expected and observed p values in blood sample EWAS. **c** delta beta (Δ β) values by lymphoid and myeloid fraction in blood samples.
