# Supplementary material for: Cigarette Smoking and E-cigarette Use Induce Shared DNA Methylation Changes Linked to Carcinogenesis
Source: Cancer Res. 2024 Mar 19;84(11):1898–914. doi: 10.1158/0008-5472.CAN-23-2957 (PMC11148547; doi:10.1158/0008-5472.CAN-23-2957)
Supplement: Figure S4 — Supplementary Figure 4 [file can-23-2957_figure_s4_suppsf4.pdf]

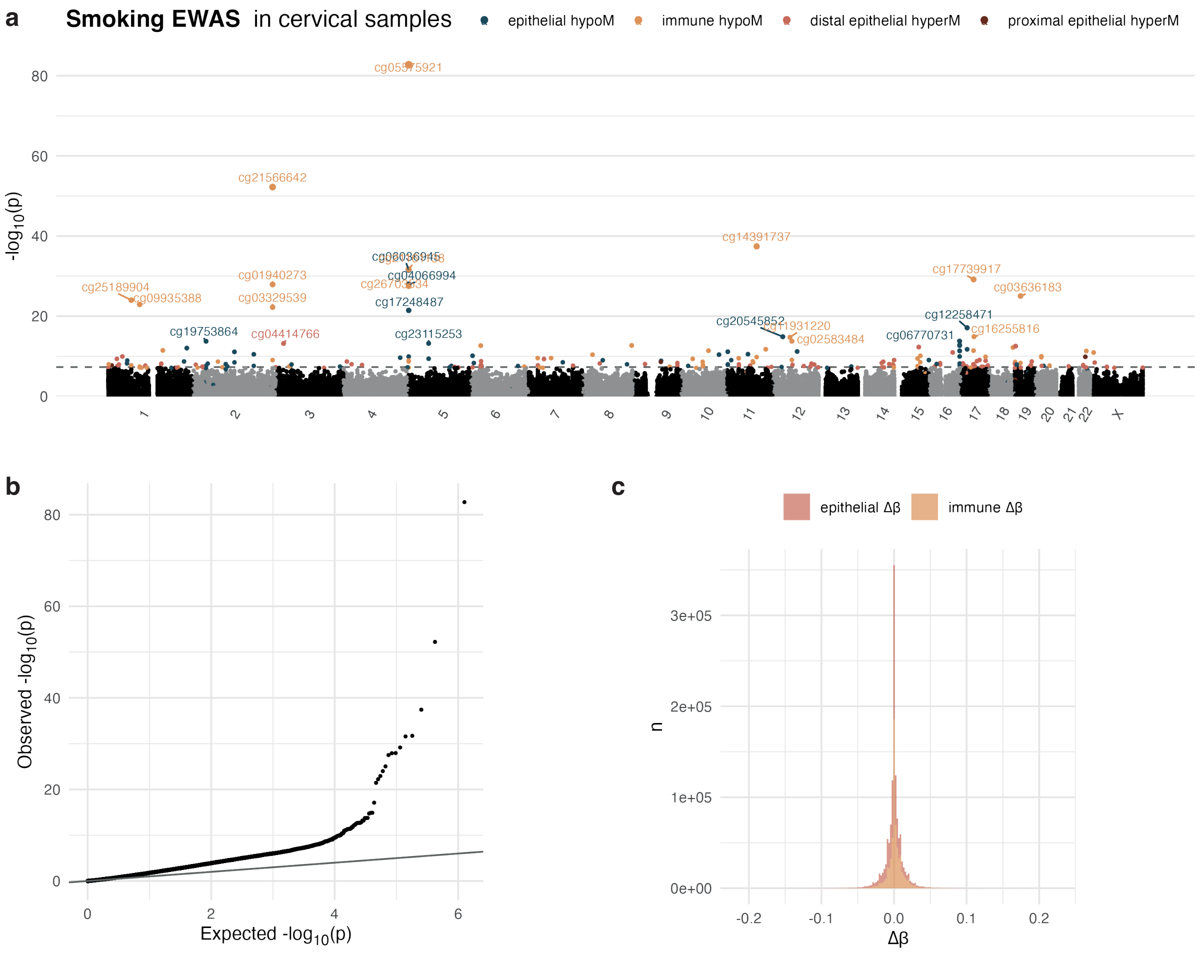

Supplementary Figure 4. Manhattan and qq-plots for smoking-associated CpGs in cervical samples after accounting for age and immune cell proportion. a Manhattan plot for smoking EWAS in cervical samples. CpGs were considered significant if they passed Bonferroni correction (equivalent to p <7.9e-08). **b** q-q plot for expected and observed p values in cervical sample EWAS. **c** delta beta (Δ β) values by epithelial and immune fraction in cervical samples.
