# Supplementary material for: Cigarette Smoking and E-cigarette Use Induce Shared DNA Methylation Changes Linked to Carcinogenesis
Source: Cancer Res. 2024 Mar 19;84(11):1898–914. doi: 10.1158/0008-5472.CAN-23-2957 (PMC11148547; doi:10.1158/0008-5472.CAN-23-2957)
Supplement: Figure S5 — Supplementary Figure 5 [file can-23-2957_figure_s5_suppsf5.pdf]

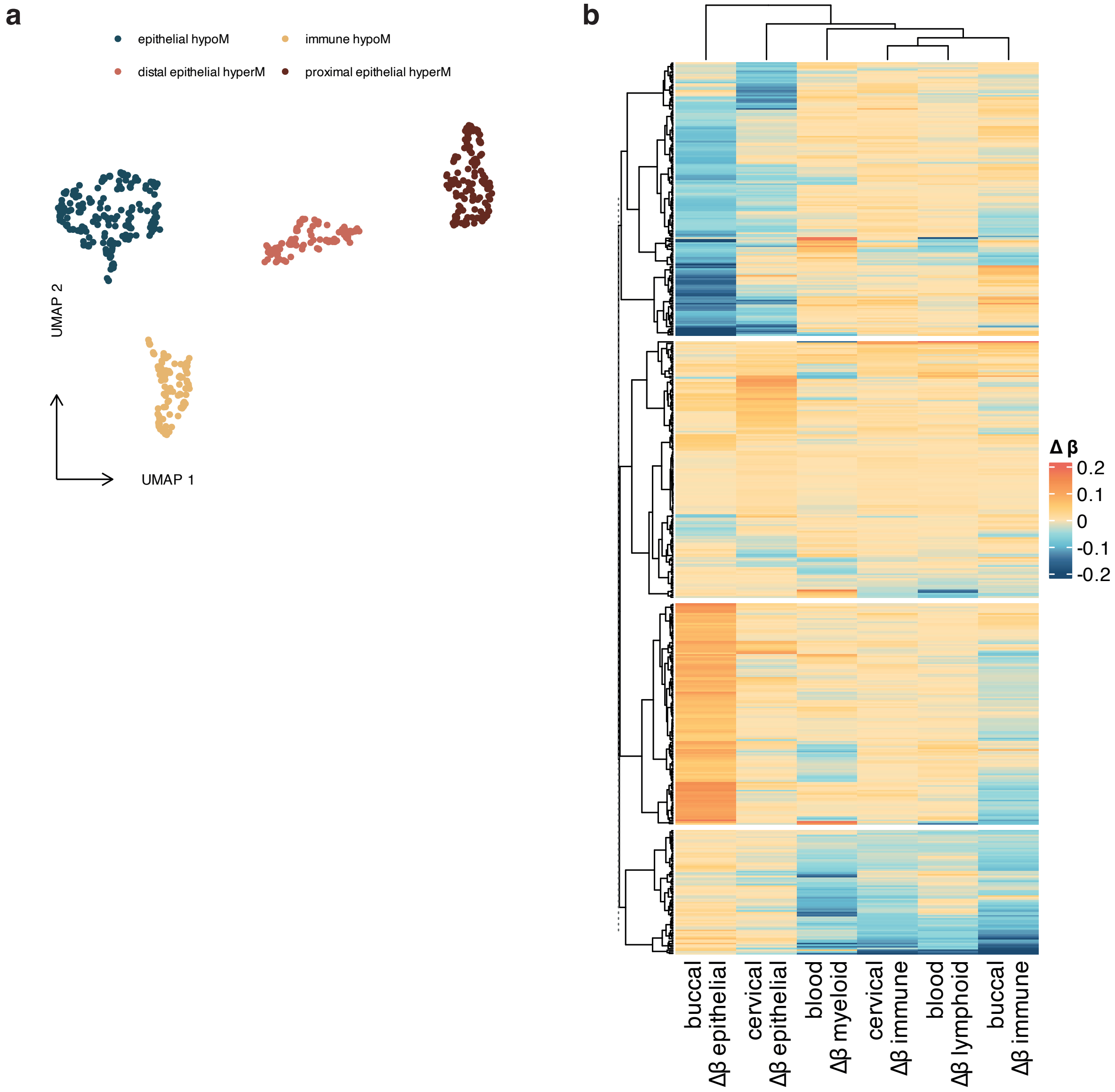

Supplementary Figure 5. Clustering results for cell-specific delta-beta values of significant CpGs using two different approaches. a Uniform manifold approximation and projection (UMAP) of the delta-beta of CpGs significantly associated with smoking in at least one tissue indicates the existence of four clusters. Clusters identified here are visualised in Figure 1c and named based on differential methylation in specific tissues and cell types. **b** A second, independent distance-based clustering, utilizing Manhattan distance and Ward's D based on the delta-beta matrix of CpGs significantly associated with smoking in at least one tissue reveals similar clustering as shown in textbf{a} (and Figure 1c).
