# Supplementary material for: Cigarette Smoking and E-cigarette Use Induce Shared DNA Methylation Changes Linked to Carcinogenesis
Source: Cancer Res. 2024 Mar 19;84(11):1898–914. doi: 10.1158/0008-5472.CAN-23-2957 (PMC11148547; doi:10.1158/0008-5472.CAN-23-2957)
Supplement: Figure S6 — Supplementary Figure 6 [file can-23-2957_figure_s6_suppsf6.pdf]

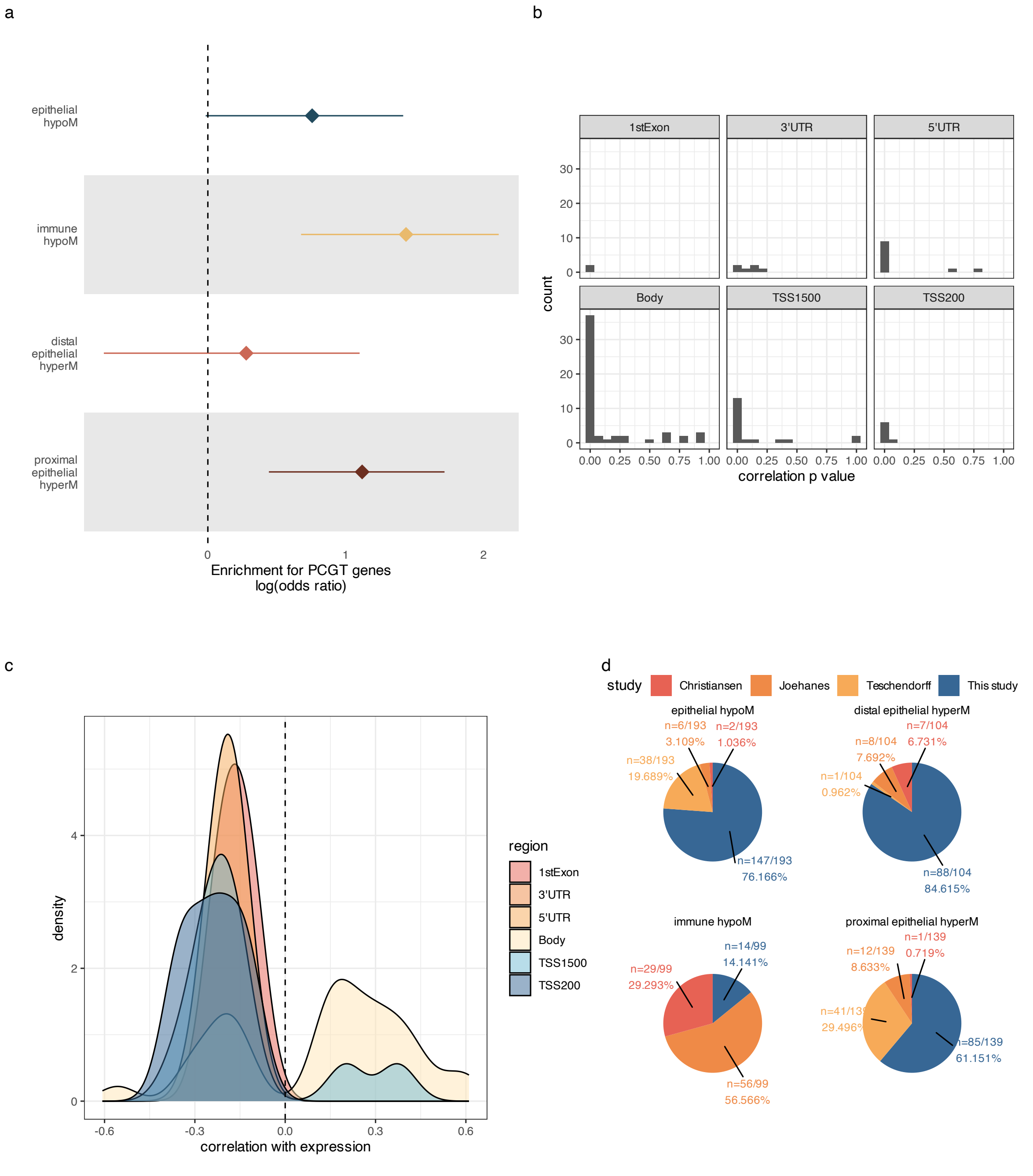

Supplementary Figure 6. Polycomb group target enrichment in CpGs in the four sets, association with expression, and overlap with previously identified sites. **a** Enrichment for Polycomb group target genes amongst CpGs in the different sets. **b** Histogram of p values for correlation of CpG loci with matched gene expression in TCGA-LUAD and TCGA-LUSC samples, based on genomic location of the CpG. **c** Density plot showing the association of CpG methylation with gene expression by genomic region. Only CpGs that were significantly correlated with expression after Bonferroni correction were included (p <0.05, 55/98). **d** Overlap of CpGs with previously identified smoking CpGs in studies by Joehanes et al., Teschendorff et

al., or Christiansen et al.
