# Supplementary material for: Cigarette Smoking and E-cigarette Use Induce Shared DNA Methylation Changes Linked to Carcinogenesis
Source: Cancer Res. 2024 Mar 19;84(11):1898–914. doi: 10.1158/0008-5472.CAN-23-2957 (PMC11148547; doi:10.1158/0008-5472.CAN-23-2957)
Supplement: Figure S7 — Supplementary Figure 7 [file can-23-2957_figure_s7_suppsf7.pdf]

a

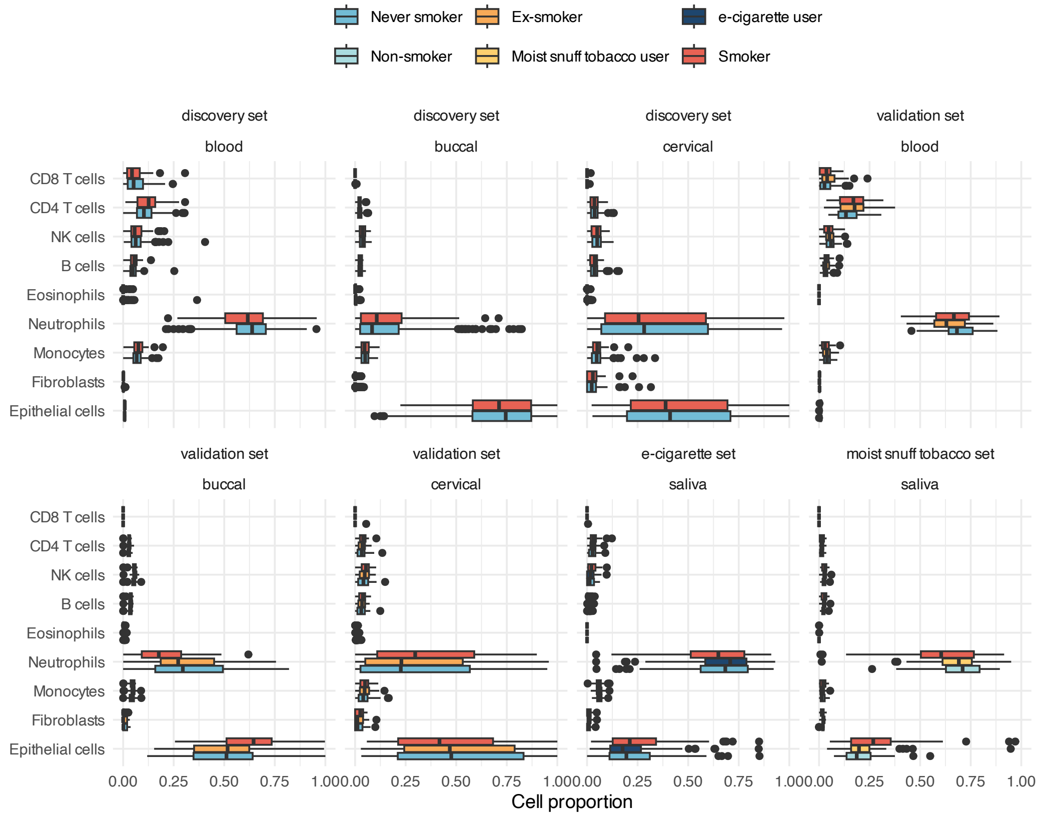

b

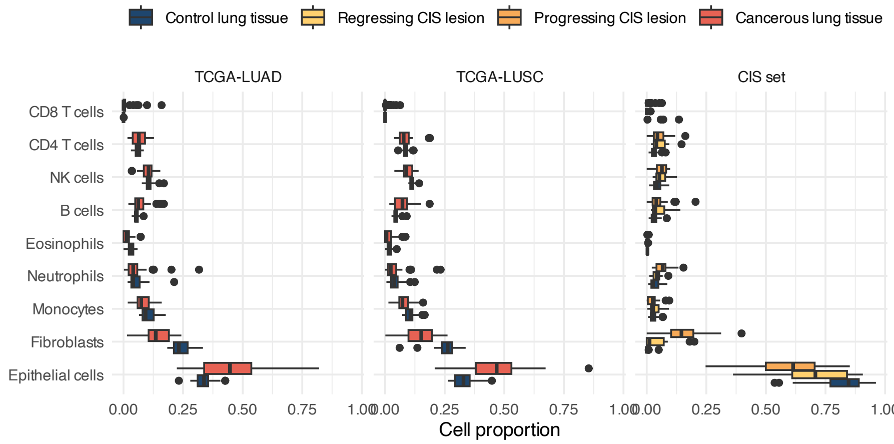

Supplementary Figure 7. Epigenetically inferred cell type composition in datasets used in this study. **a** Inferred cell type proportions in surrogate samples (buccal, blood, cervical) in various datasets included in this study. **b** Inferred cell type proportions in lung tissue datasets. Values were inferred using the EpiDISH package and reference matrices centEpiFibIC.m, and additionally, hierarchical EpiDISH (hEpiDISH) was applied using the centBloodSub.m reference matrix.

Abbreviations: TCGA, The Cancer Genome Atlas. LUAD, lung adenocarcinoma. LUSC, lung squamous cell carcinoma. CIS, carcinoma in situ.
