# Supplementary material for: Cigarette Smoking and E-cigarette Use Induce Shared DNA Methylation Changes Linked to Carcinogenesis
Source: Cancer Res. 2024 Mar 19;84(11):1898–914. doi: 10.1158/0008-5472.CAN-23-2957 (PMC11148547; doi:10.1158/0008-5472.CAN-23-2957)
Supplement: Figure S8 — Supplementary Figure 8 [file can-23-2957_figure_s8_suppsf8.pdf]

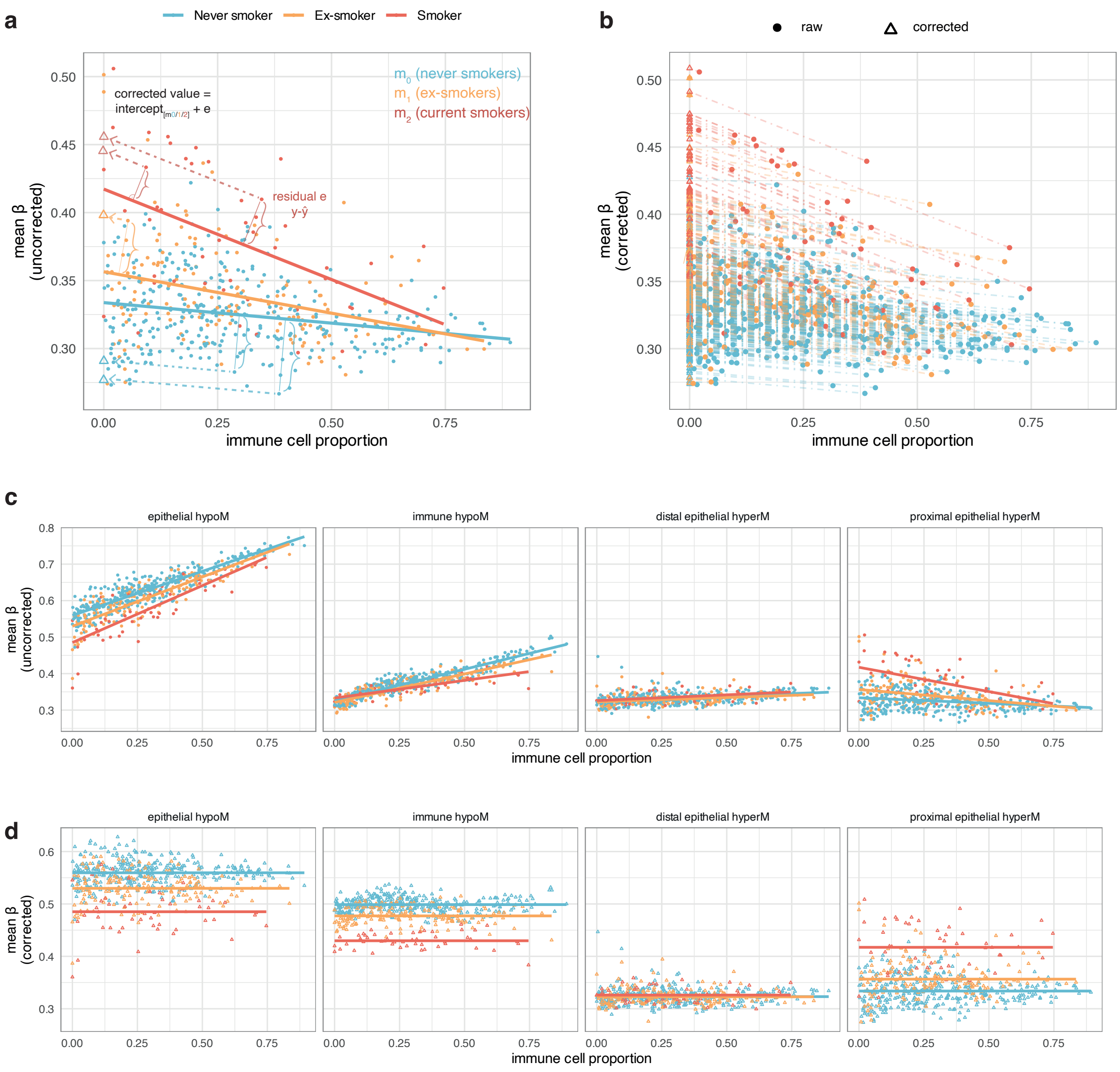

Supplementary Figure 8. Correction of mean beta values for cell type heterogeneity. a Raw mean beta values of proximal epithelial hyperM CpGs (450k) in the discovery set buccal samples. The correction approach is based on slopes and residuals for never, ex- and current smokers by fitting individual linear models for each type. **b** Visualisation of corrected mean beta of values in a, projecting values to immune cell proportion = 0. **c** Raw mean methylation values of all four groups of CpGs in buccal samples in the discovery set. **d** Corrected mean methylation values of all four groups of CpGs in buccal samples in the discovery set.
