# Supplementary material for: Cigarette Smoking and E-cigarette Use Induce Shared DNA Methylation Changes Linked to Carcinogenesis
Source: Cancer Res. 2024 Mar 19;84(11):1898–914. doi: 10.1158/0008-5472.CAN-23-2957 (PMC11148547; doi:10.1158/0008-5472.CAN-23-2957)
Supplement: Figure S9 — Supplementary Figure 9 [file can-23-2957_figure_s9_suppsf9.pdf]

a

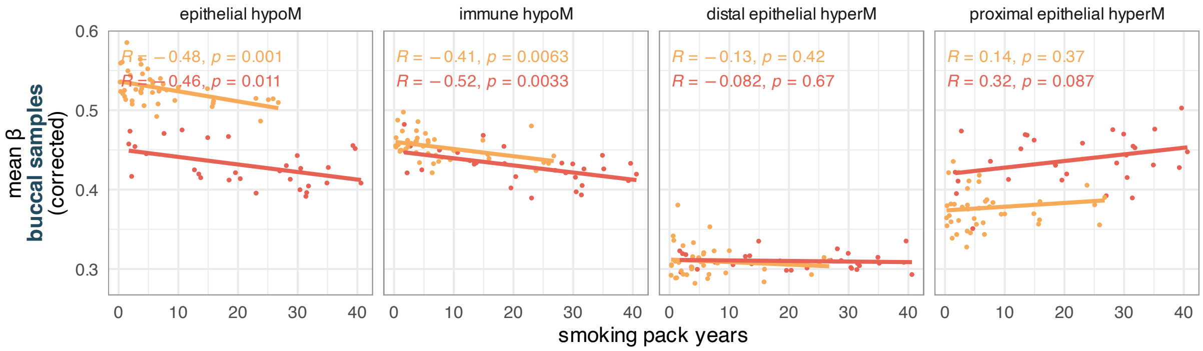

b

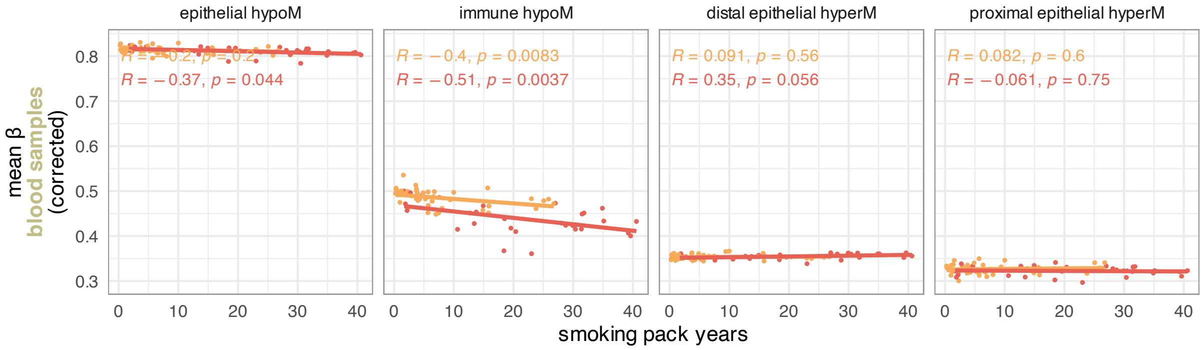

Supplementary Figure 9. Correlation of corrected mean beta values with smoking pack years (buccal and blood samples). For buccal and blood samples, smoking pack year information was available. **a** Pearson correlation of corrected mean beta values of the four groups of CpGs with smoking pack years in buccal samples. **b** Pearson correlation of corrected mean beta values of the four groups of CpGs with smoking pack years in buccal samples.
