# Supplementary material for: Cigarette Smoking and E-cigarette Use Induce Shared DNA Methylation Changes Linked to Carcinogenesis
Source: Cancer Res. 2024 Mar 19;84(11):1898–914. doi: 10.1158/0008-5472.CAN-23-2957 (PMC11148547; doi:10.1158/0008-5472.CAN-23-2957)
Supplement: Figure S10 — Supplementary Figure 10 [file can-23-2957_figure_s10_suppsf10.pdf]

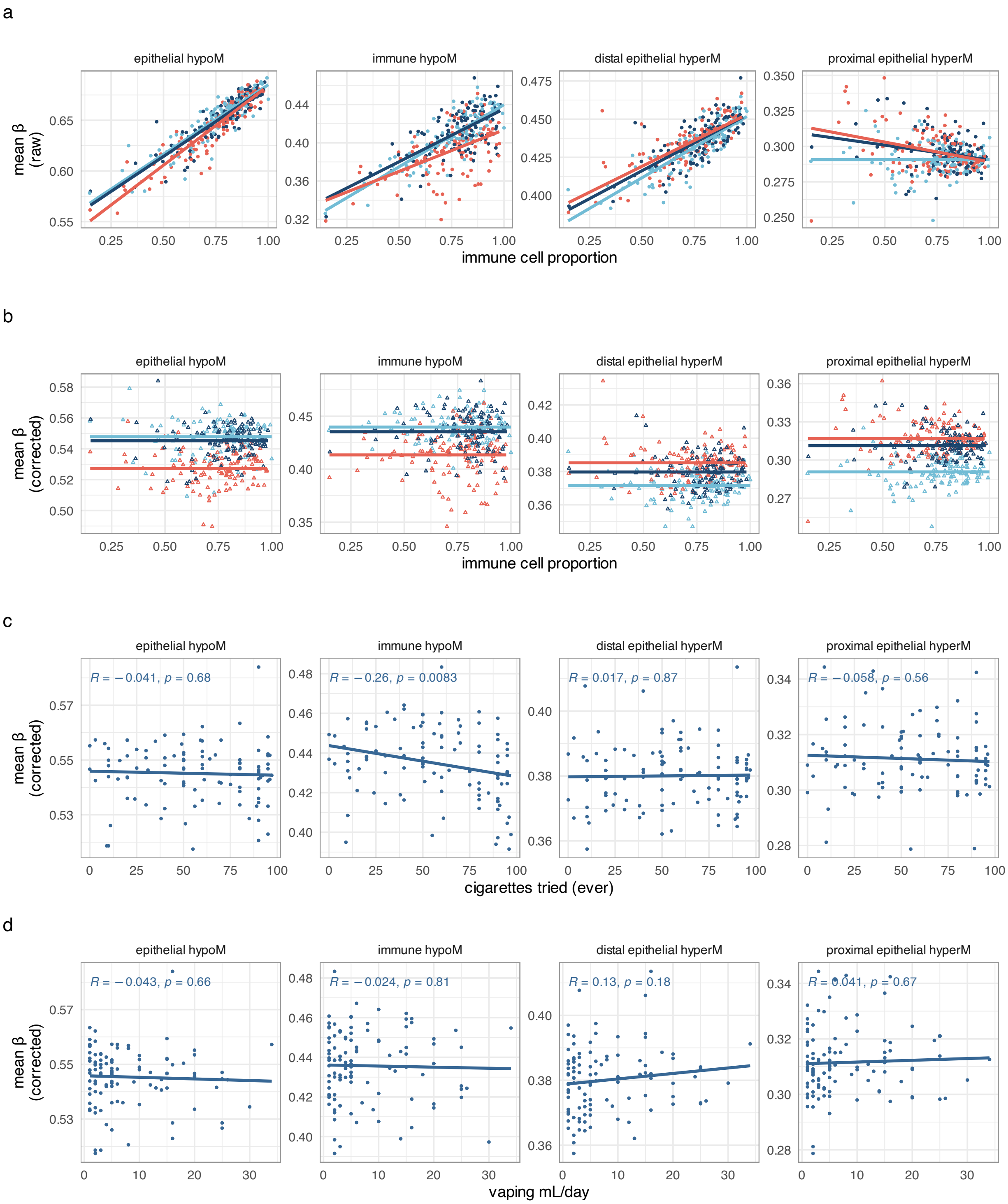

Supplementary Figure 10. E-cigarette dataset raw and corrected methylation values and correlation of corrected scores with cigarette and e-cigarette use. **a** Raw methylation mean beta values for each group of CpGs. **b** Corrected methylation mean beta values in the same samples. **c** Correlation of methylation beta values and reported number of cigarettes tried (ever) by e-cigarette users. **d** Correlation of methylation beta values and reported mL/day use of e-cigarette liquid.
