# Supplementary material for: Cigarette Smoking and E-cigarette Use Induce Shared DNA Methylation Changes Linked to Carcinogenesis
Source: Cancer Res. 2024 Mar 19;84(11):1898–914. doi: 10.1158/0008-5472.CAN-23-2957 (PMC11148547; doi:10.1158/0008-5472.CAN-23-2957)
Supplement: Figure S11 — Supplementary Figure 11 [file can-23-2957_figure_s11_suppsf11.pdf]

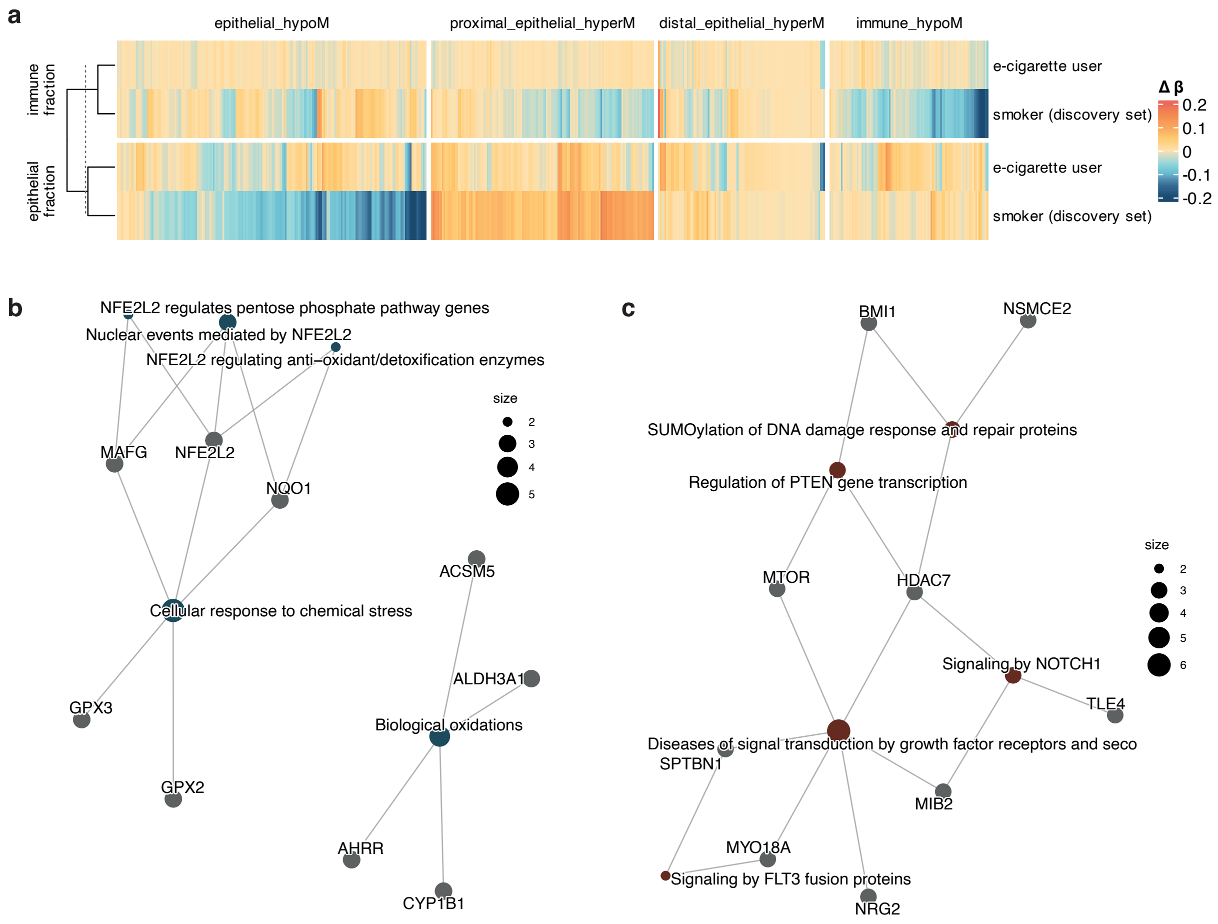

Supplementary Figure 11. E-cigarette differential methylation at individual loci and gene set enrichment. **a** Heatmap comparing delta beta values in the inferred immune and epithelial fractions in buccal samples of the discovery set and e-cigarette users. **b** Reactome pathway enrichment for sites sharing the same directionality in the epithelial fraction of epithelial hypoM as in smokers in the discovery set. **c** Reactome pathway enrichment for sites sharing the same directionality in the epithelial fraction of proximal epithelial hyperM as in smokers in the discovery set.
