# Supplementary material for: Cigarette Smoking and E-cigarette Use Induce Shared DNA Methylation Changes Linked to Carcinogenesis
Source: Cancer Res. 2024 Mar 19;84(11):1898–914. doi: 10.1158/0008-5472.CAN-23-2957 (PMC11148547; doi:10.1158/0008-5472.CAN-23-2957)
Supplement: Figure S12 — Supplementary Figure 12 [file can-23-2957_figure_s12_suppsf12.pdf]

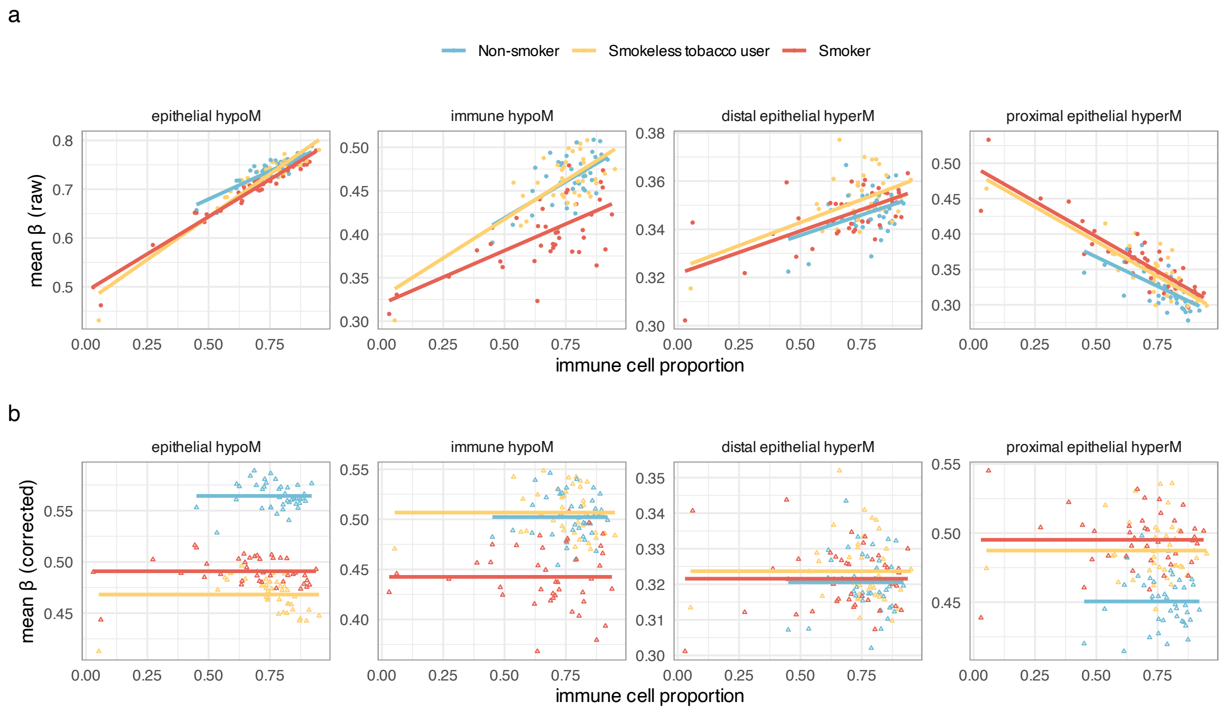

Supplementary Figure 12. Smokeless tobacco use dataset raw and corrected methylation values. a Raw methylation mean beta values for each set of CpGs. **b** Corrected methylation mean beta values in the same samples.
