# Supplementary material for: Cigarette Smoking and E-cigarette Use Induce Shared DNA Methylation Changes Linked to Carcinogenesis
Source: Cancer Res. 2024 Mar 19;84(11):1898–914. doi: 10.1158/0008-5472.CAN-23-2957 (PMC11148547; doi:10.1158/0008-5472.CAN-23-2957)
Supplement: Figure S13 — Supplementary Figure 13 [file can-23-2957_figure_s13_suppsf13.pdf]

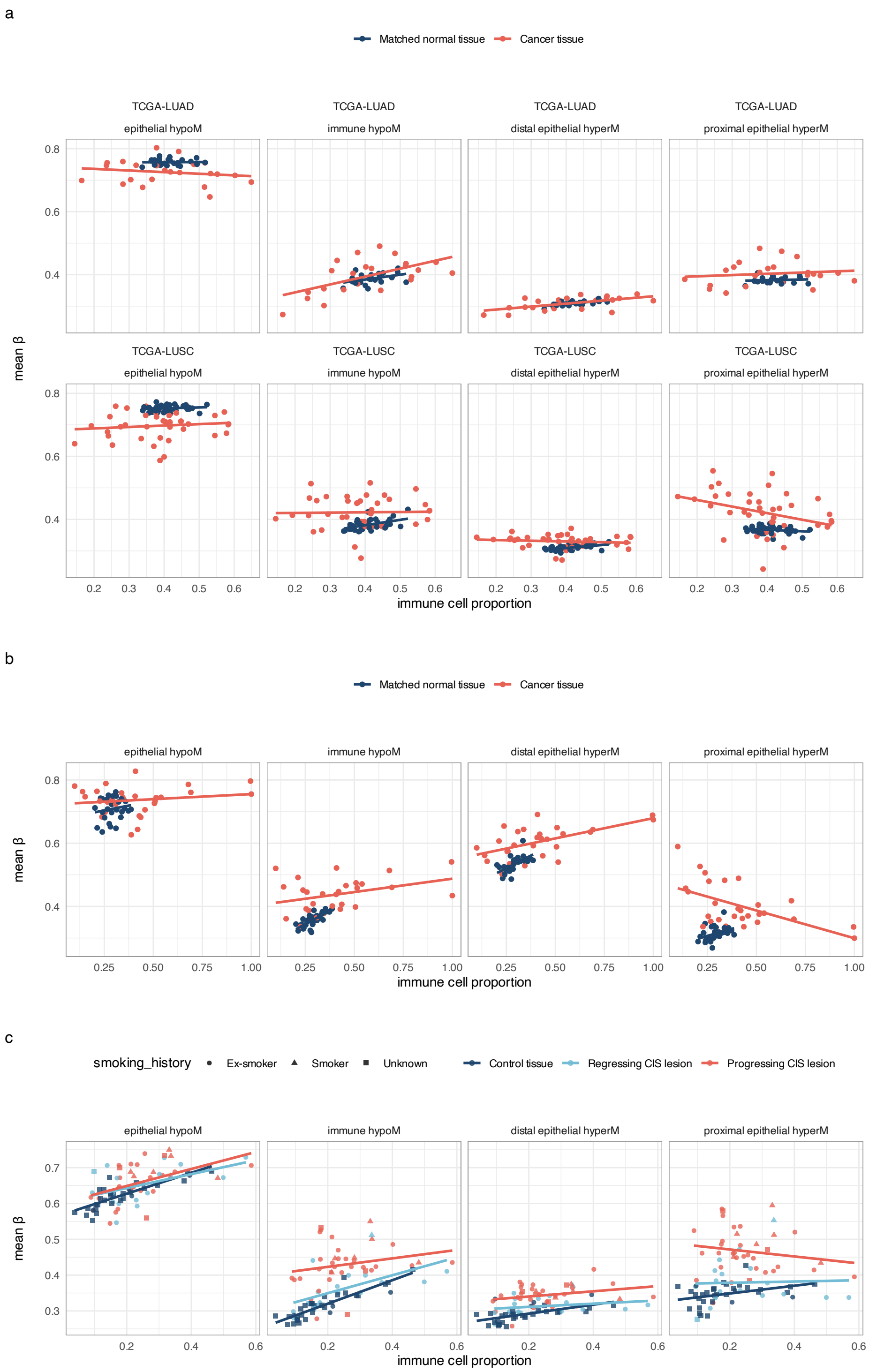

Supplementary Figure 13. Dependence of mean methylation values on immune cell composition in cancer tissue and carcinoma in situ lesions. a Raw methylation mean beta values for each set of CpGs versus inferred immune cell proportion in TCGA-LUAD and TCGA-LUSC samples with matched control tissue. **b** Raw methylation mean beta values for each set of CpGs versus inferred immune cell proportion

in cervical cancer versus normal control tissue. **c** Raw methylation mean beta values for each set of CpGs in control samples or regressing or progressing CIS lesions.
